# *Hes1* regulates anagen initiation and hair follicle regeneration through modulation of hedgehog signaling

**DOI:** 10.1002/stem.3117

**Published:** 2019-11-26

**Authors:** Wei‐Jeng Suen, Shao‐Ting Li, Liang‐Tung Yang

**Affiliations:** ^1^ Institute of Cellular and System Medicine National Health Research Institutes Zhunan Taiwan R.O.C.; ^2^ Graduate Institute of Biomedical Sciences China Medical University Taiwan R.O.C.

**Keywords:** adult stem cells, cellular proliferation, epidermis, notch, signal transduction

## Abstract

Adult hair follicles undergo repeated cycling of regression (catagen), resting (telogen), and growth (anagen), which is maintained by hair follicle stem cells (HFSCs). The mechanism underlying hair growth initiation and HFSC maintenance is not fully understood. Here, by epithelial deletion of *Hes1*, a major Notch downstream transcriptional repressor, we found that hair growth is retarded, but the hair cycle progresses normally. *Hes1* is specifically upregulated in the lower bulge/HG during anagen initiation. Accordingly, loss of *Hes1* results in delayed activation of the secondary hair germ (HG) and shortened anagen phase. This developmental delay causes reduced hair shaft length but not identity changes in follicular lineages. Remarkably, *Hes1* ablation results in impaired hair regeneration upon repetitive depilation. Microarray gene profiling on HFSCs indicates that *Hes1* modulates Shh responsiveness in anagen initiation. Using primary keratinocyte cultures, we demonstrated that *Hes1* deletion negatively influences ciliogenesis and Smoothened ciliary accumulation upon Shh treatment. Furthermore, transient application of Smoothened agonist during repetitive depilation can rescue anagen initiation and HFSC self‐renewal in *Hes1*‐deficient hair follicles. We reveal a critical function of *Hes1* in potentiating Shh signaling in anagen initiation, which allows sufficient signaling strength to expand the HG and replenish HFSCs to maintain the hair cycle homeostasis.

## Graphical abstract

Working model: By modulating ciliary function, Hes1 potentiates Shh signaling in anagen initiation, which allows sufficient signaling strength to expand the hair germ and replenish HFSCs to maintain the hair cycle.

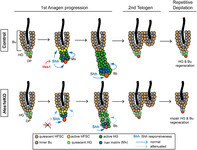

**Significance statement**

The adult hair follicles cycle through regression, resting, and growth phases, which is maintained by hair follicle stem cells. During hair growth, progenitors and stem cells of the hair follicle are activated to sustain the downward growth of hair follicles. The understanding of progenitor activation and stem cell maintenance during the hair cycle is still not complete. The present study uncovers a potential link between Notch/Hes1 and Sonic Hedgehog pathways, in which *Hes1* reinforces Hedgehog signaling at the onset of hair growth to expand the progenitors and replenish the stem cells to maintain the hair cycle homeostasis.

## 1 INTRODUCTION

Adult stem cells maintain tissue homeostasis and regeneration throughout an animal's lifetime. The murine hair follicle (HF) provides a model system for the mechanistic study of stem cell behavior during tissue regeneration. The HF consists of three regions: the lower segment (bulb), middle segment (bulge and isthmus), and upper segment (infundibulum). After initial morphogenesis, the lower segment of HFs undergoes repeated cycles of regression (catagen), resting (telogen), and growth (anagen) phases. Underpinning this regenerative cycle is the multipotent and self‐renewal capability of hair follicle stem cells (HFSCs), which reside in a specialized niche called the bulge.^1^

In telogen the bulge HFSCs and secondary hair germ (HG), a small cluster of founder cells beneath the bulge, are kept quiescent through actively repressive signals coming from the niche components and extrafollicular environment.^2^ Counteracting regulatory pathways which include activating Wnt signaling and inhibitory BMP signaling are involved in hair growth. At anagen onset, the HG becomes activated prior to bulge HFSCs by responding to BMP inhibitors and Wnt activators produced by the dermal papillae (DP), a population of mesenchymal cells that directly adjoins the HG, as well as the surrounding macroenvironment. The progeny of proliferative HG then expands downward and generates the hair matrix (Mx). The HG‐derived transit‐amplifying cells (TACs) in the Mx rapidly proliferate and differentiate into the hair shaft and inner root sheath (IRS) during anagen. To sustain anagen progression, TACs in early anagen secrete Shh to promote bulge HFSC proliferation and to stimulate dermal factors to support TAC expansion.^3^ In catagen, the hair progeny (Mx, lower ORS) undergoes apoptosis and the remaining epithelial strand retracts upward together with the DP. At the catagen/telogen transition, some slow‐cycling upper ORS cells survive after catagen to become the new bulge/HG and fuel the next hair cycle.^4^, ^5^, ^6^

Notch signaling involves ligand‐receptor interactions between contacting cells, leading to serial proteolysis of the Notch receptor. This generates the Notch intracellular domain that translocates into the nucleus where it binds Rbpj and Mastermind to activate downstream effectors, including the *Hes* and *Hey* gene families of transcriptional repressors.^7^ Loss and gain‐of‐function animal studies revealed that the canonical Notch‐Rbpj signaling axis acts as a commitment switch at the basal/suprabasal layer of the epidermis.^8^ Loss of Notch signaling does not affect HF patterning or hair placode formation; however, it was shown that HF terminal differentiation requires Notch activity.^8^, ^9^ Whether Notch signaling plays a role in HFSC activation and HF cycling remains elusive, since ablation of Notch1 in HFs causes smaller hair bulb and premature catagen entry.^10^, ^11^

The basic helix‐loop‐helix gene *Hes1* is an important effector mediating context‐dependent functions of Notch signaling in a variety of tissue types. *Hes1* maintains the stem/progenitor cells in the nervous and digestive systems by negatively regulating tissue‐specific basic helix‐loop‐helix activators.^12^ Moreover, *Hes1* is expressed in spinous keratinocytes and keeps their progenitor fate during epidermal development.^13^ Interestingly, the *Hes1*‐null epidermis developed normally when transplanted to adult mice, suggesting a restricted role of *Hes1* in developmental stages. Although *Hes1* is expressed at low levels in telogen HFs, its expression is increased in growing HFs.^14^ As a major Notch downstream effector, the role of *Hes1* in HF differentiation and regenerative hair cycling remains unclear.

Hedgehog signaling is initiated by hedgehog ligands (Sonic Hedgehog, Indian Hedgehog, and Desert Hedgehog) binding to Patched receptor, which derepresses and allows accumulation of Smoothened (Smo) in the primary cilium.^15^ Smo activation transmits downstream signaling cascade to Gli family zinc finger transcription factors, which govern Hedgehog target gene expression. The Hedgehog signaling pathway functions in both the epithelium and mesenchyme during HF development.^16^ Studies in Sonic Hedgehog (Shh) conventional knockout mice reveal that Shh signaling is dispensable for HF initial morphogenesis but required for HF down‐growth in the maturation phase. The smaller DP developed in Shh knockout mice also suggested that Shh is required for DP maintenance.^17^, ^18^ Hedgehog signaling controls numerous developmental processes in a duration‐ and intensity‐dependent manner.^19^

We have demonstrated previously that ablation of *Pofut1*, a critical component of Notch signaling, in HF lineages resulted in disrupted telogen‐anagen transition.^20^ *Pofut1*‐deficient HFs turn into cysts at the second hair cycle, which prevented studying how the Notch‐Hes1 axis participates in hair cycle homeostasis. In this study, we inactivate *Hes1* in the skin using the K14‐Cre driver and describe a novel role for *Hes1* in regulating anagen initiation and HF regeneration through modulation of Shh responsiveness.

## 2 MATERIALS AND METHODS

### 2.1 Animals

Generation of floxed *Hes1* (*Hes1* ^fx/fx^) has been described previously.^21^ *Hes1* ^fx/fx^ mice were in ICR background and back‐crossed to C57bl/6 for 3 generations. Both Rosa26 Cre reporter and K14‐Cre mice were obtained from the Jackson laboratory (Bar Harbor, ME) and maintained in C57bl/6 background. *Hes1* ^fx/f*x*^ mice were crossed with K14‐Cre mice to generate heterozygous K14‐Cre^+/wt^;*Hes1* ^fx/wt^ mice and followed by crossing with *Hes1* ^fx/f*x*^ mice to create *Hes1* ^fx/f*x*^;K14‐Cre conditional knockout (*Hes1*eKO) mice. Age‐ and gender‐ matched littermate controls (*Hes1* ^fx/f*x*^ or *Hes1* ^fx/wt^) were used for comparison. Genotyping was performed on tail biopsies by PCR. For depilation experiments, back skin of the anesthetized mice was shaved and depilated mechanically using the Wax Strip Kit (VIGILL Pharma. Co., Taiwan). For Smoothened agonist (SAG, Santa Cruz, Dallas, Texas) rescue experiments, mice were topically applied with 25 μL vehicle (95% acetone/5% dimethyl sulfoxide) and SAG (120 μM) at opposite sides of the dorsal skin daily for consecutive 6 days after depilation. For intradermal delivery of growth factors, Affi‐Gel Blue gel beads (Bio‐Rad, Hercules, California) were coated with recombinant Shh‐N (2 μg/mouse, R&D, Minneapolis, MN) or 0.1% bovine serum albumin control and intradermally injected in the dorsal skin of mice (8‐ to 11‐week‐old) as previously described.^22^ The skins were harvested 4 days later for histological analyses.

All animal works were carried out at the research laboratory of National Health Research Institutes (NHRI) and conducted according to Taiwan COA national guidelines. All studies and procedures were performed with protocols approved by the NHRI Animal Care and Use Committee.

### 2.2 Histological analysis and immunostaining

Lower back skin samples were fixed with 4% paraformaldehyde for either 30 minutes on ice or 4 hours at room temperature, followed by frozen and paraffin embedding, respectively. All samples were sagittally sectioned at 6 μm. Hematoxylin and eosin staining and LacZ staining were performed using the standard procedures. To measure alkaline phosphatase activity in the DP, air‐dried cryostat sections were prepared, fixed in acetone for 10 minutes, and incubated with NBT/BCIP substrate (Promega, Madison, WI) following the manufacturer's instruction.

Immunochemistry and immunofluorescence staining were performed as previously described.^20^ Images were acquired with Olympus BX51 microscope equipped with Olympus DP71 CCD using DP controller and DP manager software or with a Leica TCS SP5 confocal microscope system with Leica Power 3D software. The sources and dilutions of primary antibodies were Hes1 (1:100, Santa Cruz or Cell Signaling, Danvers, MA), K6 (1:100, Thermo Fisher, Waltham, MA), AE15 (1:100, Santa Cruz), AE13 (1:100, Abcam, Cambridge, MA), K73 (1:150, Biorbyt, San Francisco, CA), K82 (1:100, Abnova, Taiwan), Ki67 (1:100, Thermo Fisher), CD34 (1:100, eBioscience, Waltham, MA), Sox9 (1:100, Santa Cruz), NFATc1 (1:150, Santa Cruz), β‐catenin (1:100, BD, San Jose, CA), P‐cadherin (1:250, R&D), p‐Smad 1/5/8 (1:1000, Santa Cruz), phospho‐histone H3 (1:100, Cell Signaling), Igfbp3 (1:100, R&D), Arl13b (1:200, Abcam), Pericentrin (1:500, Convance, Cambridge, MA), Smo (1:300, Abcam), K14 (1:250, Thermo Fisher), K15 (1:200, Thermo Fisher), Versican (1:50, Chemicon, Temecula, CA), and Vimentin (1:200, Abcam). Hes1 immunostaining was amplified by TSA Plus Cyanine 3 detection kit (PerkinElmer, Hopkinton, MA) following the manufacturer's protocol.

### 2.3 Mouse epidermal keratinocyte culture

Primary keratinocytes were isolated from the back skin of newborn mice as previously described.^23^ For cilia staining, primary keratinocytes were starved 24 hours in E‐media+0.1% chelexed‐FBS for ciliated cell enrichment. Cells were treated with vehicle, 10 nM Shh‐N (R&D), or 10 nM SAG for an additional 4 hours (immunostaining) or 16 hours (qRT‐PCR).

### 2.4 FACS and flow cytometry

Isolation of HFSCs based on α6‐integrin and CD34 were performed following the published protocol.^24^ In brief, telogen dorsal skin with dermal adipose removed with scalpel was treated with dispase (5 U/mL, Invitrogen, Waltham, MA) in Hanks' balanced saline solution at 4°C overnight, and then transferred to Trypsin‐ethylenediaminetetraacetic acid (0.25%, Invitrogen) at 37°C for 10 minutes. The resulting single cell suspension was filtered through a 70‐μm cell strainer and incubated with CD49f‐PE and biotinylated‐CD34 antibodies followed by streptavidin‐APC. Cell sorting was done on a FACS Influx cell sorter equipped with FACS Software (BD, Franklin Lakes, NJ). Keratinocytes with high forward and side scatter as well as dead cells (7‐AAD+) were gated out, and the HFSCs (CD34+CD49f+) were collected. Flow cytometry were performed on a FACSCalibur analyzer (BD) and data were analyzed with the FlowJo program.

### 2.5 RNAscope in situ hybridization

RNAscope in situ hybridization was performed following the manufacturer's protocol (Advanced Cell Diagnostics, Newark, CA). The RNAscope probes used are Mm_*Gli1* (311001), Mm_*Ptch1* (402811), and Mm_*Hes1* (417701). Each sample was quality controlled for RNA integrity using a positive control RNAscope probe Mm_*Ppib* (313911) and a negative control probe bacteria dapB (310043).

### 2.6 Data acquisition and statistics

For immunostaining, identical conditions of exposure and background balance for image capture were used for comparisons between control and mutant samples. Positively stained cells were counted manually in a defined area of the tissues. Image J software (NIH) was used to measure the length and pixel intensity in photos for quantification study. Statistical analyses were done using either a Student's *t*‐test for comparing two samples or an analysis of variance followed by Tukey's multiple comparisons test for comparing multiple samples. *P*‐value less than .05 was considered to be significant.

## 3 RESULTS

### 3.1 Ablation of notch signaling effector *Hes1* in the murine epidermis causes retarded hair growth

**Figure 1:**
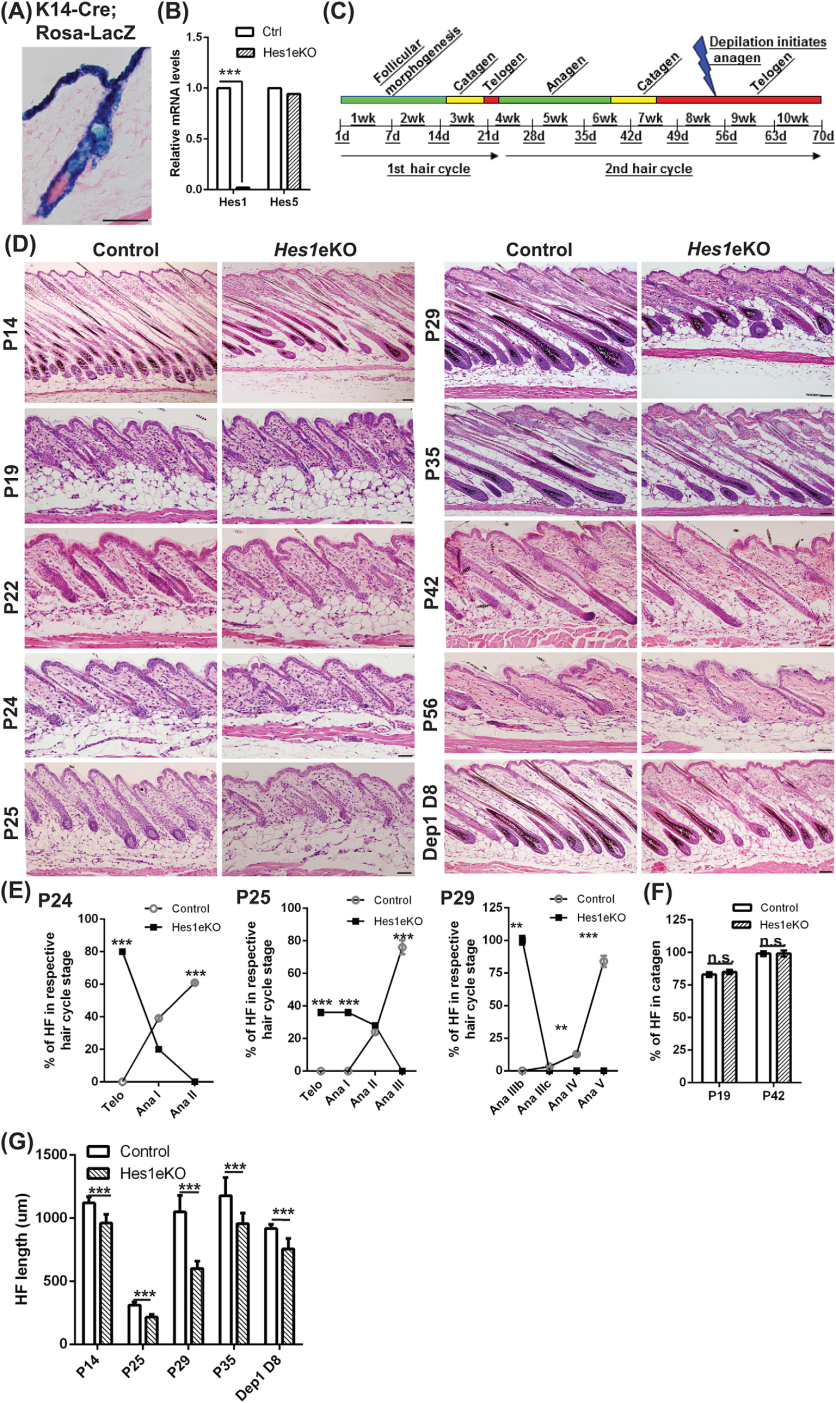
*Hes1* deletion using K14‐Cre deleter line causes retarded hair growth. A, X‐gal‐stained back skin section of [K14‐Cre^+/wt^; Rosa‐LacZ ^+/wt^] mice with eosin counterstain. B, Quantitative real‐time PCR (qRT‐PCR) analysis of *Hes1* and *Hes5* expression levels in the back skin epithelium of *Hes1*eKO and control mice (mean ± SD, n = 4, ***P < .001). C, Illustration of the two synchronous hair cycles coordinated with age in mice after birth. D, Hematoxylin and eosin‐stained back skin sections at P14 (follicular morphogenesis), P19 (first catagen), P22 (first telogen), P24 (second early anagen), P25 (second anagen), P29 (second anagen), P35 (second late‐anagen/early catagen), P42 (second catagen), P56 (second telogen), and day 8 post‐depilation at P56. E and F, Quantification of hair cycle stage according to morphology‐based hair cycle histomorphometry^25^ in each hair cycle phase as indicated (mean ± SD, n > 50 hair follicles (HFs) per genotype from two to three independent control and mutant pairs at each phase, n.s., nonsignificant; **P < .01; ***P < .001). G, Quantification of HF length (from the base of follicle to the follicular orifice) in each hair cycle phase as indicated (mean ± SD, n > 50 HFs per genotype from two to three independent control and mutant pairs at each phase, ***P < .001). Scale bar, 50 μm

We explored the function of *Hes1*, a major Notch downstream target, in the epidermis and HFs using a conditional knockout study. We crossed the *Hes1* fx/fx mice to K14‐Cre mice, and the resulting [*Hes1* ^fxfx^;K14‐Cre] mice (hereafter referred to as *Hes1*eKO mice) were born without any overt phenotype. We used the surrogate Rosa26‐LacZ reporter mice to confirm K14‐Cre‐induced gene recombination in the entire postnatal epidermis (Figure 1A). Quantitative real‐time PCR (qRT‐PCR) of *Hes1* and *Hes5*, two major Notch effectors in the epidermis, revealed that *Hes1* gene expression is significantly decreased in the *Hes1*eKO epidermis whereas *Hes5* is unaffected (Figure 1B).

We examined the gross phenotype of the back skin during the postnatal hair cycle and found that the anagen progression was significantly delayed in *Hes1*eKO mice (Figures 1C and S1A). In histological and quantitative analyses (Figure 1D, G), *Hes1*eKO HFs were shorter than control HFs in follicular morphogenesis (P14), but catagen induction was similar to control HFs at P19. The telogen to anagen transition was delayed in *Hes1*eKO HFs, since fewer HFs were in advanced anagen phase during P24 to P29 (Figure 1E).^25^ *Hes1*eKO HFs were shorter than control HFs during the second anagen (P25‐P35, Figure 1G). The anagen‐catagen transition (P35‐P42) as well as catagen‐telogen transition (P42‐P56) were comparable between *Hes1*eKO and control HFs. Plucking of telogen HFs stimulates anagen re‐entry,^25^ and the HFs of *Hes1*eKO mice were shorter than control mice 8 days post‐depilation at P56 (Figure 1G). These data suggested that *Hes1*eKO HFs displayed retarded hair growth during homeostasis and depilation‐induced hair regeneration.

### 3.2 *Hes1*‐deficient HFs display delayed anagen initiation and shortened hair growth phase

**Figure 2:**
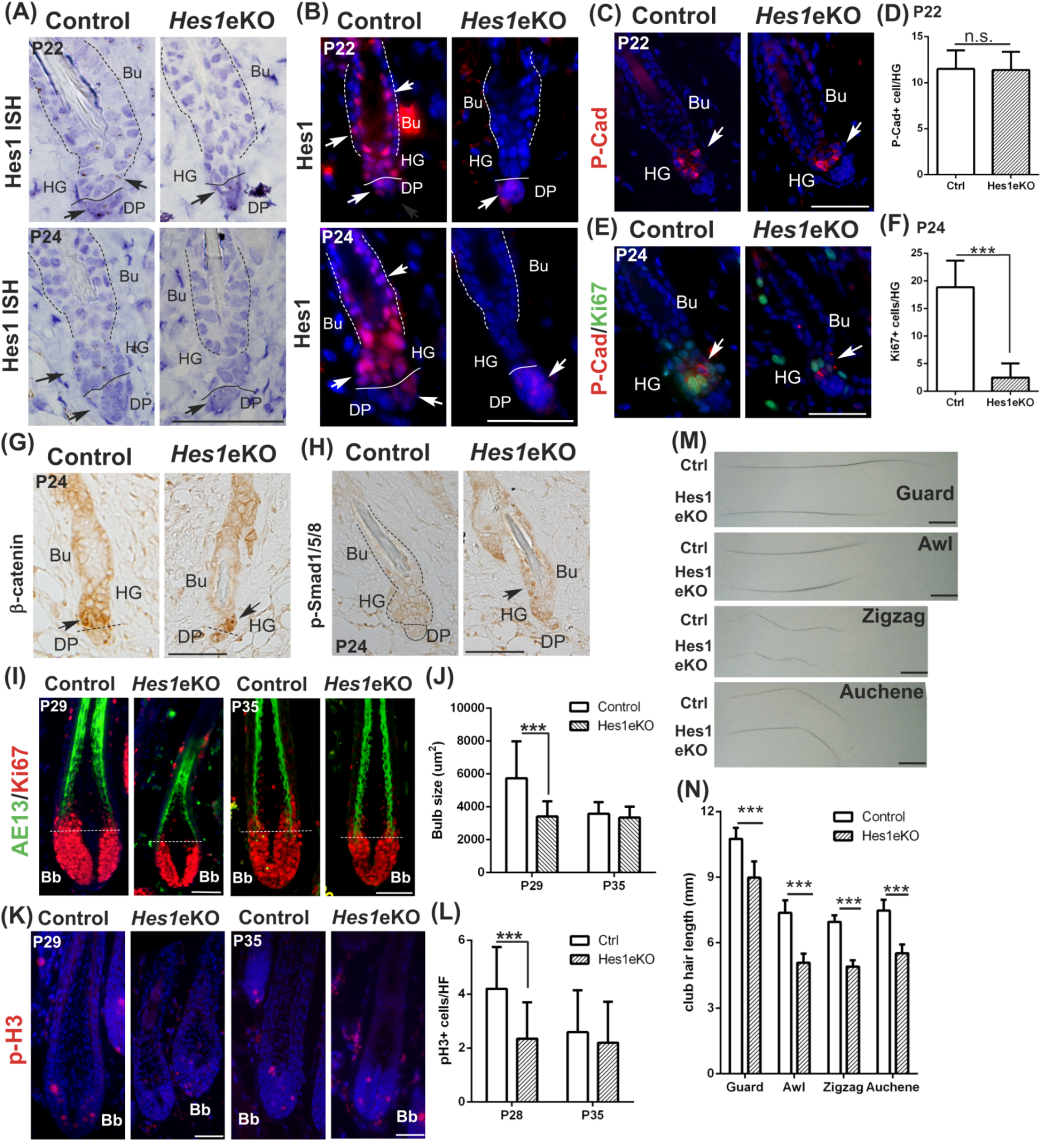
*Hes1* deletion results in delayed anagen initiation and shortened hair follicle (HF) growth phase. A, In situ hybridization of *Hes1* (arrows) in back skin sections at P22 and P24 with hematoxylin counterstain. The dotted lines demarcate the bulge and the solid lines demarcate the boundary between DP and HG when visible. B, Back skin sections were immunostained for Hes1 at P22 and P24. C, Back skin sections were immunostained for HG marker P‐Cadherin (P‐Cad, arrows) at P22. D, Quantification of P‐Cad + cells in the HF at P22 (mean ± SD, n > 30 HFs per genotype from three independent control and mutant pairs, n.s., nonsignificant). E, Back skin sections were double immunostained for P‐Cad and proliferative marker Ki67 (arrows) at P24. F, Quantification of Ki67+ cells in the P‐Cad + cells at P24 (mean ± SD, n > 30 HFs per genotype from three independent control and mutant pairs, ***P < .001). G, Back skin sections at P24 were immunostained for β‐catenin. The arrows mark the nuclear β‐catenin staining. The dotted lines denote the boundary between DP and HG when visible. H, Back skin sections immunostained for phospho‐Smad1/5/8 (arrows). The dotted line marks the HF and solid line marks the DP. I, Double immunostaining of AE13 and KI67 in back skin sections at P29 (full anagen) and P35 (late anagen/early catagen). The dotted lines depict the line of Auber. J, Quantification of the bulb size (Ki67+ area below the line of Auber) (mean ± SD, >40 HFs from three biological replicates per genotype per stage, ***P < .001). K, Back skin sections immunostained for cell mitotic marker phospho‐histone H3 (p‐H3) at P29 and P35. L, Quantification of p‐H3+ cells in the hair matrix (mean ± SD, >40 HFs from three biological replicates per genotype per stage, ***P < .001). DAPI counterstaining in blue. Bu, bulge; HG, hair germ; DP, derma papillae; Bb, hair bulb. M, Bright field images of club hair of four different hair types at P60. N, Quantification of club hair length of each HF type (mean ± SD, 20 club hairs for each hair types per mouse, n = 3 biological replicates per genotype, ***P < .001 determined by analysis of variance). Scale bar, 50 μm except (M), where it is 1 mm

Using in situ hybridization and immunostaining, we demonstrated that *Hes1* is expressed in the bulge and enriched in the lower bulge/HG during anagen initiation. Although *Hes1* expression was detected in the inner bulge and less frequent in the outer bulge layers in telogen, it was detected in both the inner and outer bulge layer during anagen initiation. *Hes1* expression was absent in the HF epidermal compartment of *Hes1*eKO HFs, whereas that in the DP remained (Figure 2A, B). Next, we analyzed anagen initiation by immunostaining of P‐cadherin (HG marker) and Ki67 (proliferative marker). We found that *Hes1*eKO HFs displayed decreased cell proliferation in the HG compared with control HFs at early anagen (P24), whereas no differences in the HG cell numbers were observed at telogen (P22, Figure 2C‐2F). Generally, anagen activation is accompanied by nuclear translocation of β‐catenin, a marker of active Wnt signaling, in the HG.^26^ The β‐catenin immunostaining revealed that *Hes1*eKO HFs displayed fewer nuclear β‐catenin signals than control HFs (Figure 2G). Accordingly, control HFs displayed less phospho‐Smad1/5/8 staining, a marker of inhibitory BMP signaling, than *Hes1*eKO HFs (Figure 2H). Furthermore, given the comparable immunostaining of HFSC markers CD34, Sox9, NFATc1, and K15 (Figure S1B‐D) as well as the lack of TUNEL staining in control and *Hes1*eKO HFs (Figure S1E), we demonstrated that neither loss of HFSCs nor increased cell death in the HG accounted for the delayed anagen entry in *Hes1*eKO HFs.

To investigate the anagen progression defects in *Hes1*eKO HFs, we performed immunostaining for AE13 and Ki67 to quantify the hair bulb size (Figure 2I, J), as well as immunostaining for phospho‐histone H3, a cell mitotic marker, to quantify the matrix proliferation (Figure 2K, L). Our data indicated that the hair bulbs of *Hes1*eKO HFs were smaller and less proliferative than that of control HFs at P29 (anagen). In late anagen‐catagen transition (P35), the hair bulb size and matrix proliferation of *Hes1*eKO HFs did not exceed control HFs, suggesting that *Hes1*eKO HFs had never grown to the size as control did. We excluded increased cell death as the underlying cause for smaller hair bulbs in *Hes1*eKO HFs, as evidenced by TUNEL staining on samples harvested at the second hair cycle (P29‐P56, Figure S2A). Smaller hair bulbs and less Mx proliferation of *Notch1*‐deficient HFs have been attributed to paracrine Igfbp3 induced in the DP.^11^ However, we found no discernible difference in levels of Igfbp3 protein between control and *Hes1*eKO DPs in both anagen and telogen phases (Figure S2B). The DP characteristics and inductive ability were examined by alkaline phosphatase activity and Versican protein expression, as well as counting the number of Versican+ cells in the DP (Figure S2C‐E), and we found no difference between control and *Hes1*eKO HFs. Collectively, these data indicated that *Hes1*eKO HFs displayed delayed anagen initiation and shortened HF growth phase.

### 3.3 *Hes1* deletion causes reduced hair shaft length but not identity changes in follicular lineages

*Hes1* is expressed in the Mx, precortex, medulla, cortex, and cuticle of the hair shaft,^14^ which implicates its function in HF differentiation. To examine whether *Hes1* deficiency causes any hair structure defect (Figure S2F), we analyzed the hair keratin markers K6, AE15, AE13, K82, and K73 at P29 (anagen) and P35 (late anagen). Although K6 staining revealed that both control and *Hes1*eKO HFs have comparable companion layers (Figure S2G), immunostaining of other markers revealed that *Hes1*eKO HFs lack the hair shaft medulla layer (AE15) and exhibited less developed hair shaft (AE13) and cuticle layers in both the IRS (K73) and hair shaft (K82) at P29. Remarkably, hair shaft AE15+ medulla layer and the AE13+, K82+, and K73+ cell layers of *Hes1*eKO HFs appeared to be comparable to control HFs at P35 (Figure S2H). These data indicated that *Hes1* deletion caused delayed follicular lineage formation but not identity changes.

Mouse hair coat consists of four different HF types (Guard, Awl, Zigzag, Auchene) that emerge in three waves during development.^27^ We found that *Hes1*eKO mice have all four HF types; however, the club hair length of four HF types is shorter in *Hes1*eKO mice than in control mice at P60 (Figure 2M, N). We conclude that the shortened anagen phase resulted from *Hes1* deletion causes reduced hair shaft length.

### 3.4 *Hes1* is required for HF regeneration in a sequential depilation model

**Figure 3:**
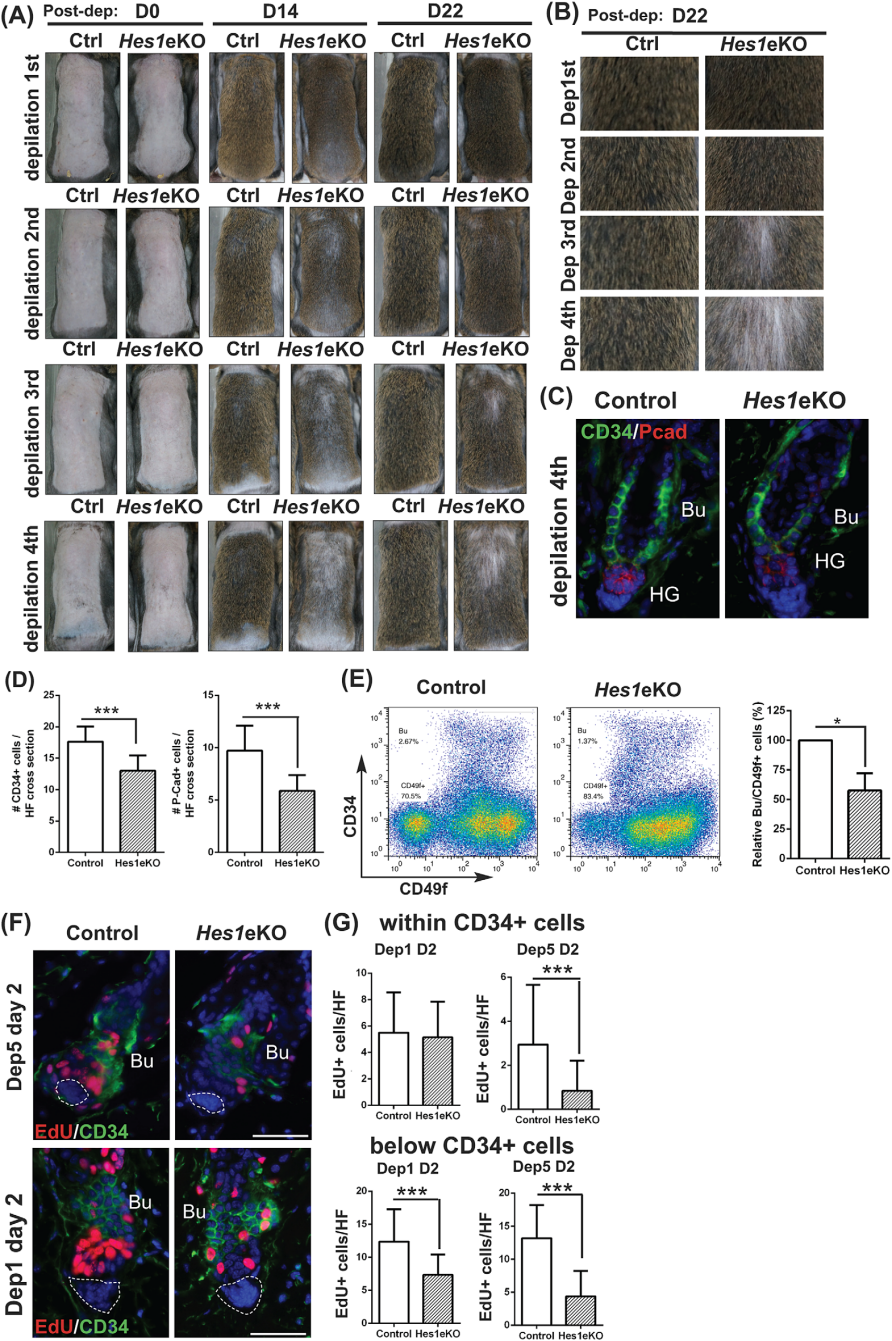
*Hes1* deficiency causes compromised hair follicle (HF) regeneration and hair follicle stem cell (HFSC) self‐renewal after repetitive depilation. A, Sequential depilation of control littermate (Ctrl) and *Hes1* conditional knockout (*Hes1*eKO) mice for four rounds with a three‐week interval from the second telogen. Representative pictures of male mice are shown (n = 5). B, Close up of back skin at day 22 post‐depilation‐induced hair regeneration. C, Back skin sections from repetitive depilation (day 22 after fourth depilation) were double immunostained for CD34 and P‐Cad. D, Quantification of CD34+ bulge and P‐Cad + HG cells in HFs after four rounds of sequential depilation (mean ± SD, n > 50 HFs per genotype from four independent control and mutant pairs, ***P < .001). E, Quantifications of HFSCs after four rounds of sequential depilation using flow cytometry. Data are presented as percentage of Bu/CD49f + cells relative to control samples (mean ± SD, n = 4 independent pairs). F, Skin sections from sequential depilation (one and five times) were processed for CD34 immunostaining and EdU incorporation assays. Dotted lines denote the DP when visible. G, Quantification of cell proliferation within CD34+ cells and below CD34+ cells after sequential depilation (mean ± SD, n > 50 HFs per genotype from three independent control and mutant pairs for each set, ***P < .001). Bu, Bulge; HG, hair germ; SG, sebaceous glands. DAPI counterstaining in blue. Scale bar, 50 μm

To assess the function of *Hes1* in regenerative hair cycle, we applied a repetitive depilation model to induce HFSC activation and monitoring the HF regeneration. Although control mice could mostly replenish the hair coat, *Hes1*eKO mice displayed a gradual thinning of hair coat after repetitive depilation (Figures 3A, B, and S3A, B). We observed gender difference in HF regeneration; male *Hes1*eKO mice displayed hair coat thinning early than female *Hes1*eKO mice. Immunostaining of CD34 and P‐Cadherin revealed that HFSCs and HG cells were reduced in *Hes1*eKO mice after repetitive depilation (Figures 3C, D, and S3C, D). Using flow cytometry to quantify the HFSCs, we found a significant reduction in HFSC population in *Hes1*eKO mice after repetitive depilation (Figure 3E). We applied CD34 immunostaining and EdU incorporation assays to examine the HFSC activation after repetitive depilation. Although HFSC activation in control and *Hes1*eKO HFs were initially similar (day 2 after first depilation), HFSC activation in *Hes1*eKO HFs were compromised after five rounds of depilation (day 2 after fifth depilation) (Figure 3F, G). We additionally found that the hair coat of unperturbed *Hes1*eKO mice was thinner than control mice at about 1 year old (Figure S3E). These data indicate that *Hes1*eKO HFSCs cannot sustain HF regeneration after repeated hair‐growth cycles.

### 3.5 Loss of *Hes1* leads to compromised Shh signaling in HFSCs

**Figure 4:**
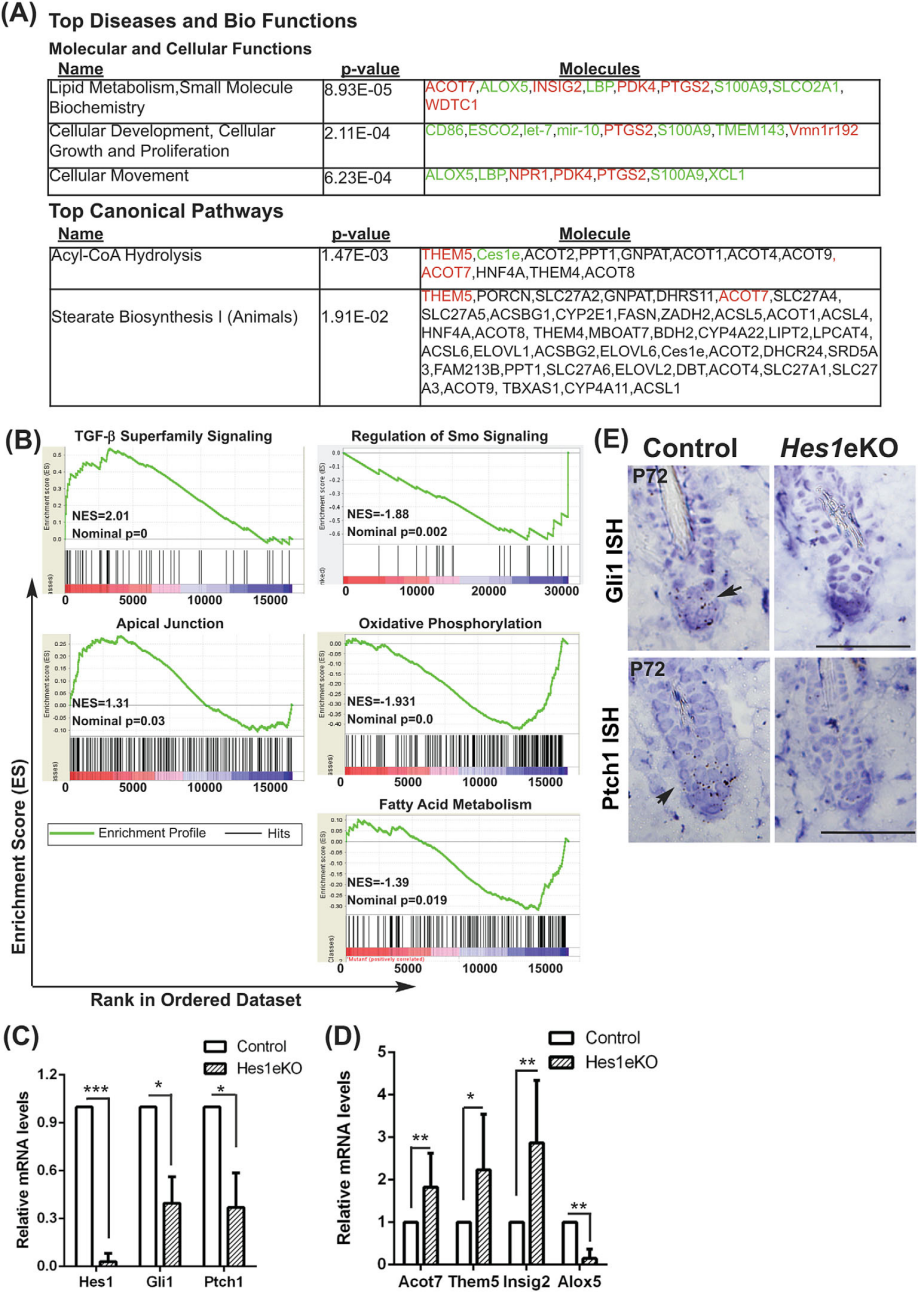
*Hes1* inactivation causes compromised Shh signaling in hair follicle stem cells (HFSCs). A, Ingenuity pathway analysis result showing the top diseases and bio functions and top canonical pathways significantly affected by *Hes1* deletion in HFSCs (cutoff fold change >1.5 or <−1.5, *P* < .05). Red and green indicate upregulated and downregulated genes in *Hes1*eKO HFSCs, respectively. B, Gene set enrichment analysis of microarray gene expression profiling on HFSCs from control and *Hes1*eKO mice (n = 2 independent pairs). C and D, qRT‐PCR analysis of selected genes related to Shh signaling, top diseases and biological functions, and top canonical pathways on FACS‐purified HFSCs from control and *Hes1*eKO mice at P72 (telogen after depilation at P50, mean ± SD, n = 4 independent pairs, *P < .05, **P < .01, ***P < .001). E, In situ hybridization of *Gli1* and *Ptch1* (arrows) in control and *Hes1*eKO back skin sections at P72 (telogen after depilation at P50). Scale bar, 50 μm

To understand the molecular basis underlying the HF phenotype in *Hes1*eKO mice, we performed microarray gene expression profiling on FACS‐purified HFSCs from control and *Hes1*eKO mice at P72 (telogen after depilation at P50). We identified 77 upregulated genes and 88 downregulated genes with a fold change >1.5 or <−1.5 (*P* < .05) in *Hes1*eKO vs control HFSCs (Figure S4A‐C). Ingenuity pathway analysis revealed “lipid metabolism,” “cellular growth and proliferation,” and “cellular movement” among the top diseases and biological functions affected by *Hes1* deletion; Acyl‐CoA hydrolysis and stearate biosynthesis are among the top canonical pathways affected by *Hes1* deletion (Figures 4A and S4D). Next, we performed gene set enrichment analysis on the microarray results. We found that the gene sets enriched in TGF‐β superfamily signaling (BMP signaling) and apical cell adhesion are specifically upregulated in *Hes1*eKO HFSCs. Remarkably, gene sets enriched in Smoothened signaling regulation, mitochondrial oxidative phosphorylation, and fatty acid metabolism are specifically downregulated in *Hes1*eKO HFSCs (Figure 4B). qRT‐PCR analyses on selected genes related to Shh signaling, top diseases and biological functions, and top canonical pathways confirmed the microarray results (Figures 4C, D, and S4E).

Indeed, the retarded anagen progression and hair regeneration failure observed in *Hes1*eKO mice closely resemble phenotypes of conditional Hedgehog component knockout mice.^3^ We therefore examined Shh signaling activity in control and *Hes1*eKO HFs at telogen, a stage when control and *Hes1*eKO HFs can be compared. In situ hybridization of the Shh target genes *Gli1* and *Ptch1* revealed decreased Shh signaling activity in *Hes1*eKO HFs at P72 (telogen after depilation at P50) (Figure 4E). Our data indicate a specific function for *Hes1* in hair cycle control through modulation of Shh signaling.

### 3.6 *Hes1* deletion causes primary cilia defect and influences hedgehog signaling responsiveness

**Figure 5:**
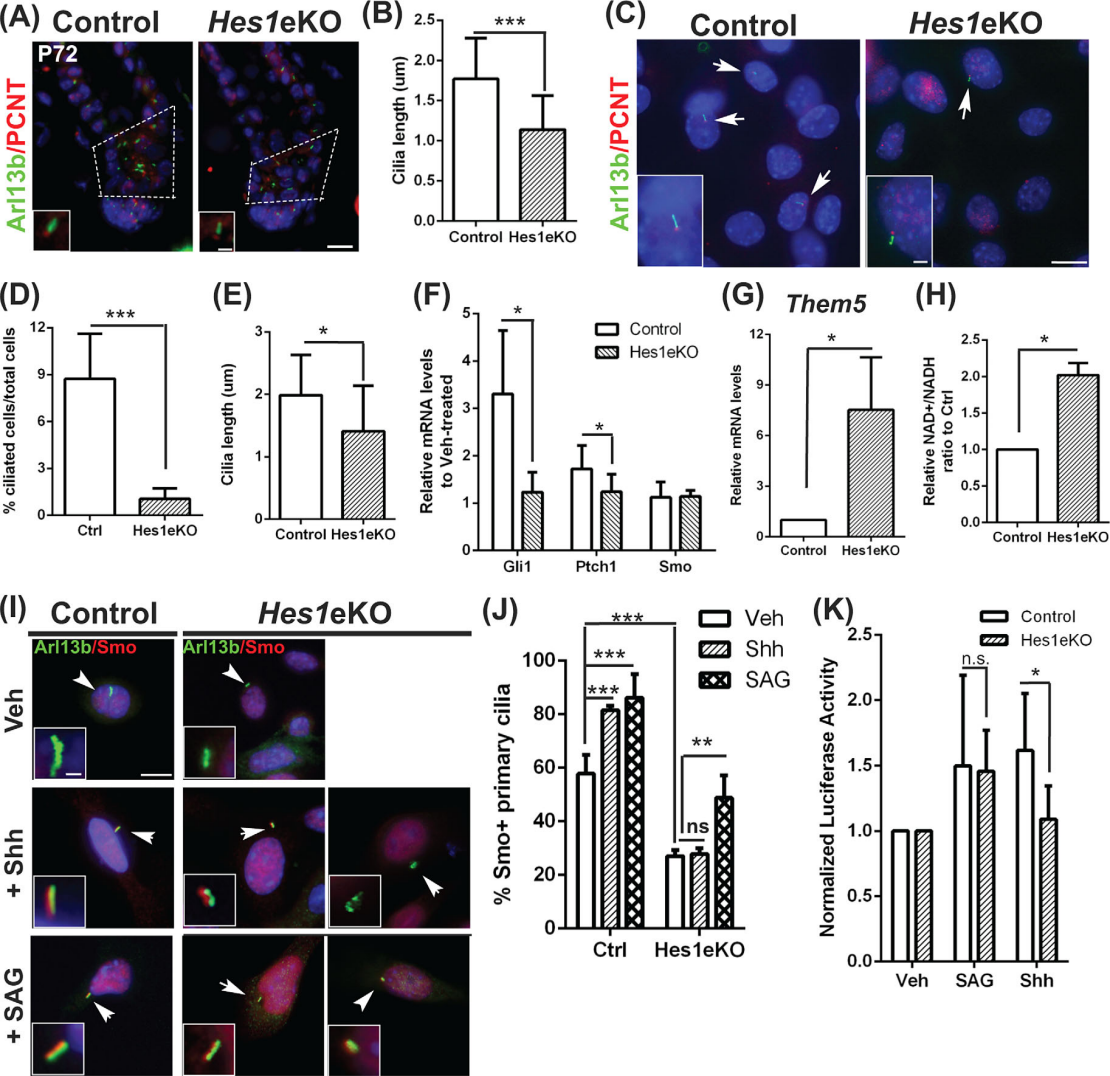
*Hes1* deletion causes primary cilia defect and influences Shh signaling responsiveness. A, Double immunostaining of Arl13b and Pericentrin in back skin sections of mice at P72 (telogen after depilation at P50). The dotted line box denotes the region of cilia measurement. The boxed region shows high magnification. DAPI counterstaining in blue. B, Quantification of cilia length in lower hair follicles (HFs) (dotted line box in (A)) of control and *Hes1*eKO mice (mean ± SD, n = 3 independent pairs; control 336 cilia; *Hes1*eKO 384 cilia, ***P < .01). C, Control and *Hes1*eKO PMEKs were immunostained for Arl13b and Pericentrin. The boxed region shows high magnification. D, Quantification of ciliated cells expressed as a percentage of total in control and *Hes1*eKO PMEKs (mean ± SD, n = 3 independent samples per genotype; >100 cells/microscopic field, >6 field/sample, ***P < .001). E, Quantification of cilia length in control and *Hes1*eKO PMEKs (mean ± SD, n = 3 independent samples per genotype; control 198 cilia; *Hes1*eKO 110 cilia, *P < .05). F, qRT‐PCR analysis of *Gli1*, *Ptch1*, and *Smo* expression levels in Shh‐treated relative to vehicle‐treated PMEKs (mean ± SD, n = 5 independent samples per genotype, *P < .05). G, qRT‐PCR analysis of *Them5* on control and *Hes1*eKO PMEKs (mean ± SD, n = 4 independent samples per genotype, *P < .05). H, Measurement of NAD^+^/NADH ratio in control and *Hes1*eKO PMEKs (mean ± SD, n = 3 independent samples per genotype; *P < .05). I, Control and *Hes1*eKO PMEKs were serum starved and treated with vehicle, Shh (10 nM), or SAG (10 nM) and double immunostained for Arl13b and Smo. The boxed region shows high magnification. Low‐ and high‐ magnification scale bars represent 10 and 1 μm. J, Quantification of the percentage of Smo + primary cilia in control and *Hes1*eKO PMEKs treated with vehicle, Shh, or SAG (mean ± SD, n = 4 independent samples per genotype per condition; control >90 cilia and *Hes1*eKO > 110 cilia in each treatment, **P < 0.01, ***P < 0.001, n.s., nonsignificant, determined by analysis of variance). K, Normalized luciferase activity in control and *Hes1*eKO PMEKs transfected with reporter plasmids and treated with vehicle, SAG, or Shh (mean ± SD, n = 4 independent samples per genotype per condition, *P < .05, n.s., nonsignificant)

Shh signaling is sensitive to the length, numbers, and architecture of primary cilia^28^ and ciliary transport of Smo is involved in Hedgehog signaling activation. We therefore examined the cilia length in the lower HFs of control and *Hes1*eKO mice by double immunostaining of Arl13b (a small GTPase localized to cilia) and Pericentrin (a centrosome protein localized to cilia base). We found that the cilia in the lower Bu/HG of *Hes1*eKO HFs were shorter than that of control HFs at P72 (Figure 5A, B). Because the ciliary accumulation of endogenous Smo was difficult to detect in tissue sections by antibody staining, we used primary mouse epidermal keratinocyte (PMEK) cultures from control and *Hes1*eKO dorsal skin as an alternative system (Figure S5). We observed a decrease in both the percentage of ciliated cells and the ciliary length in *Hes1*eKO PMEKs when cultured in serum‐starved conditions to enrich ciliated cells, as revealed by double immunostaining of Arl13b and Pericentrin (Figure 5C‐E). Accordingly, qRT‐PCR analysis revealed that *Hes1*eKO PMEKs had lower fold induction of *Gli1* and *Ptch1* mRNA than control PMEKs in response to Shh (Figure 5F). Additionally, we found increased gene expression of acyl‐CoA thioesterase *Them5* and elevated NAD/NADH ratio in *Hes1*eKO PMEKs, suggesting a correlation with altered cellular metabolism (Figure 5G, H).

Next, we examined Smo ciliary accumulation in the absence or presence of Hedgehog activators by double immunostaining of Arl13b and Smo (Figure 5I). Interestingly, we observed fewer Smo + primary cilia in *Hes1*eKO PMEKs than control PMEKs during serum starvation. The ciliary localization of Smo was increased in control PMEKs upon Shh treatment, whereas that in *Hes1*eKO PMEKs remained unchanged. In contrast, ciliary localization of Smo in *Hes1*eKO PMEKs was increased upon SAG treatment, suggesting a regulatory mechanism upstream of Smo activation (Figure 5J). Accordingly, we found that *Hes1*eKO PMEKs displayed compromised Gli binding site‐luciferase activity in response to Shh but not to SAG (Figure 5K). These findings indicate that *Hes1* modulates Shh signaling through regulation of ciliogenesis and Smo ciliary accumulation.

### 3.7 Direct activation of smoothened can rescue anagen initiation and HF regeneration in *Hes1*eKO mice

**Figure 6:**
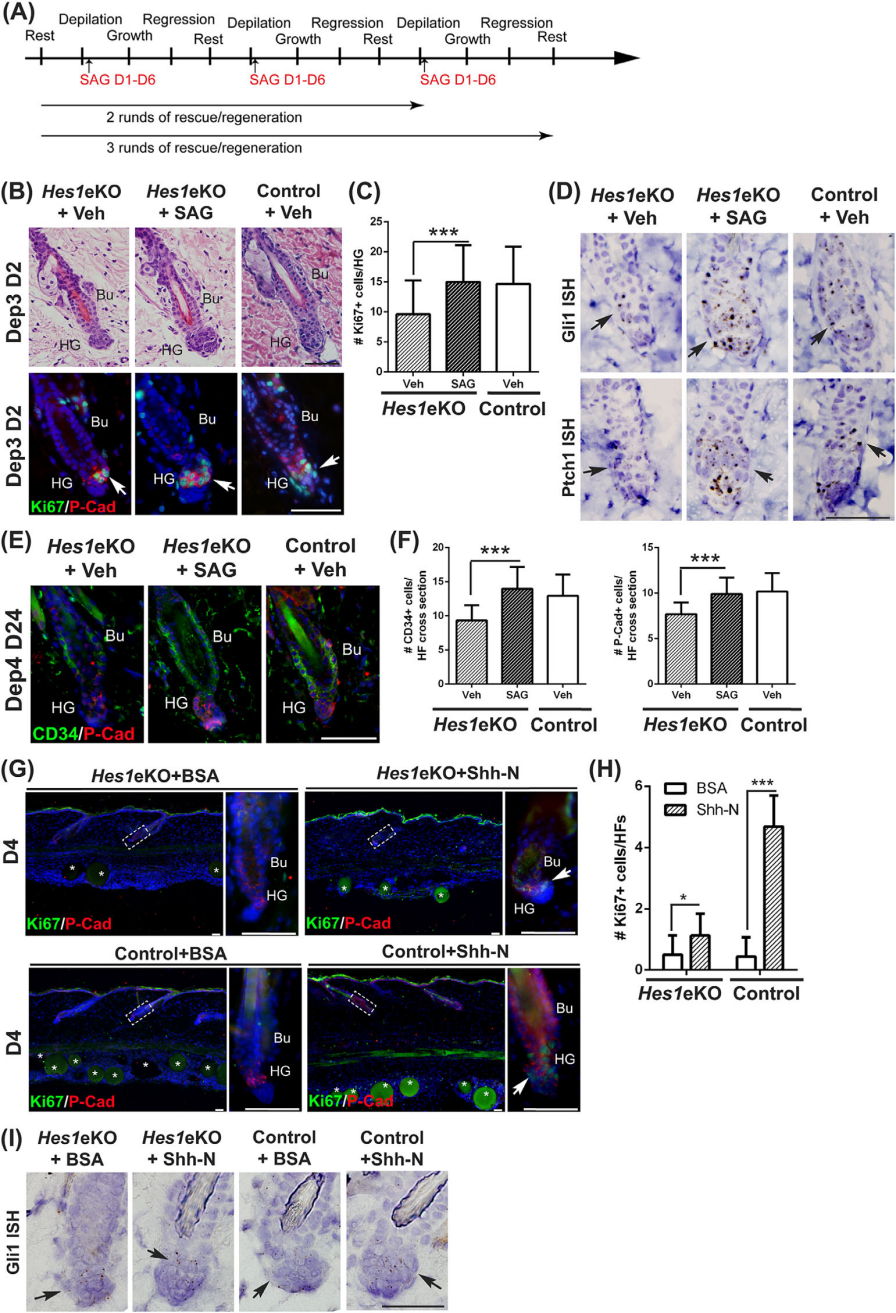
Transient application of Smoothened agonist (SAG) can rescue anagen initiation and hair follicle (HF) regeneration in *Hes1*eKO mice. A, Schematics of the in vivo SAG rescue experiments. B, Histological (hematoxylin and eosin‐stained) and immunostaining (Ki67/P‐Cad) analyses on back skin samples harvested at early anagen (day 2 after third depilation) after two rounds of depilation/SAG treatment. C, Quantification of Ki67+ cells in HG cells after SAG rescue experiments (mean ± SD, n > 50 HFs per experimental condition from three independent pairs, ***P < .001). D, In situ hybridization of *Gli1* and *Ptch1* in back skin sections from mice after two rounds of depilation/SAG treatment. E, Double immunostaining of CD34 and P‐Cad on back skin samples harvested at telogen (day 24 after fourth depilation) after three rounds of depilation/SAG treatment. F, Quantification of CD34+ bulge and P‐Cad + HG cells in HFs after SAG rescue experiments (mean ± SD, n > 40 HFs per experimental condition from three independent pairs, ***P < .001). G, Immunostaining of P‐Cad and Ki67 in skin sections of control and *Hes1*eKO mice intradermally injected with BSA or Shh‐N coated beads. Right panels, magnified views of boxed areas. Asterisks, the injected protein‐coated beads. H, Quantification of Ki67+ cells in the P‐Cad + cells from intradermal injection experiments (mean ± SD, n > 15 HFs per bead injection from three independent control and mutant pairs, *P < .05, ***P < .01). I, In situ hybridization of *Gli1* in back skin sections from bead injection experiments. Bu, bulge; HG, hair germ; DP, derma papillae. DAPI counterstaining in blue. Scale bar, 50 μm

Small molecule agonist SAG binds Smo directly and bypasses Patched receptors to activate Shh signaling. Topical application of SAG has been demonstrated to stimulate the hair regrowth in adult mouse skin.^29^, ^30^ To ascertain whether direct activation of Smo can rescue HF phenotypes in *Hes1*eKO mice, we performed transient application of vehicle and SAG at opposite sides of the back skin during repetitive depilation (Figures 6A and S6A). Although vehicle‐treated *Hes1*eKO HFs displayed anagen delay after sequential depilation, two rounds of depilation/SAG treatment rescued anagen initiation in *Hes1*eKO HFs (Figure 6B, C). In situ hybridization of *Gli1* and *Ptch1* demonstrated that Shh signaling activity in *Hes1*eKO HFs was rescued by SAG treatment (Figure 6D). After three rounds of depilation/SAG treatment, we found that both the CD34+ bulge cells and P‐Cad + HG cells were increased in *Hes1*eKO HFs (Figure 6E, F). Additionally, the club hair length of each HF types in *Hes1*eKO mice was increased by three rounds of SAG treatment (Figure S6B, S6C). To demonstrate that the Shh signaling is compromised but still functional in *Hes1*eKO HFs, we analyzed the effect of exogenous Shh administration on the back skin of control and *Hes1*eKO mice. Shh and BSA‐coated beads were intradermally injected in the dorsal skin of control and *Hes1*eKO mice. The skin sections were immunostained for P‐Cad and Ki67 as well as assayed for Gli1 mRNA expression (Figure 6G‐I). We observed that exogenous Shh administration can stimulate cell proliferation and *Gli1* mRNA induction in the HG of both control and Hes1eKO HFs, indicating that Shh signaling is functional in both control and *Hes1*eKO HFs. Our results indicate that direct stimulation of Smo activity can rescue the anagen initiation and HF regeneration in *Hes1*eKO HFs.

## 4 DISCUSSION

**Figure 7:**
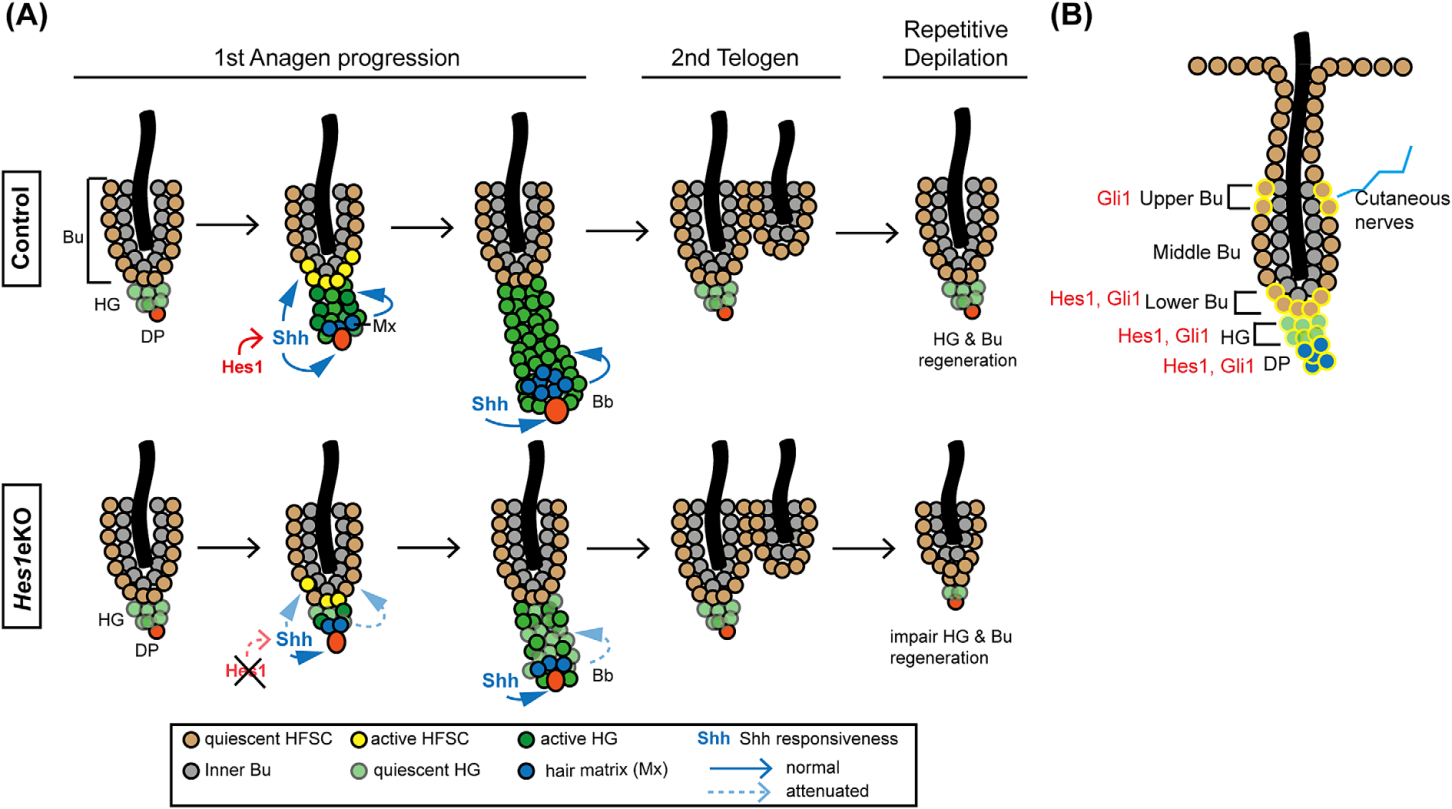
Working model of telogen‐anagen transition and hair follicle (HF) regeneration regulated by *Hes1*. A, By modulating ciliary function, *Hes1* potentiates Shh signaling in anagen initiation, which allows sufficient signaling strength to expand the hair germ and replenish hair follicle stem cells to maintain the hair cycle. B, Illustration of the location of *Hes1* and *Gli1* expression in telogen HF

The hair cycle represents a paradigm for studying stem cell quiescence and activation, as well as progenitor cell proliferation, differentiation, and death. Here, we show that *Hes1* expression is enriched in the lower bulge/HG at anagen onset. The retarded hair growth observed in *Hes1*‐deficient HFs is resulted from a delay in anagen initiation and shortened anagen phase. Moreover, *Hes1* epithelial ablation results in impaired HF regeneration after repetitive depilation. Transcriptome analysis and gene expression data indicate that *Hes1* ablation compromises Shh responsiveness. *Hes1* possibly influences Hedgehog signaling through regulating ciliogenesis and Smo ciliary accumulation. Therefore, direct activation of Smo can rescue anagen initiation and HFSC self‐renewal in *Hes1*‐deficient HFs. Our data suggest that *Hes1* reinforces the Shh signaling during telogen‐anagen transition to maintain hair cycle homeostasis (Figure 7A).

A role for Notch signaling in postnatal HF development and cycling was delineated by epithelial knockout of Notch components. Smaller hair bulbs were reported at the postnatal HF morphogenesis, and premature entry into catagen was postulated to be the underlying cause.^10^ Similar phenotype was reported by Lee et al, in which smaller hair bulb of *Notch1*‐deficient HFs was attributed to lower mitotic rates mediated by paracrine inhibition of IGF signaling in the Mx through DP‐derived IGFBP3.^11^ However, *Hes1* expression was unaltered in *Notch1*‐deficient HFs, nor did we observe any difference in *Igfbp3* expression as well as characteristics and inductive ability between control and *Hes1*eKO DPs. The delayed anagen entry observed in *Hes1*‐deficient HFs suggests a cell‐autonomous role for *Hes1* in stem cell/progenitor activation during anagen induction.

Notch ligands and receptors are expressed in the skin in a complex and dynamic manner.^31^ Notch downstream effectors are expressed in the hair bulb precortex and hair shaft precursors when the Mx commits terminal differentiation, suggesting a role for Notch signaling in hair shaft differentiation.^14^ Interestingly, we found that *Hes1*‐deficient HFs displayed a delayed occurrence of hair shaft components without changes in hair follicular lineages, suggesting that *Hes1* modulates the response of HF stem/progenitor cells to hair growth promoting signals rather than directly regulates lineage commitment.

The delayed anagen initiation could be resulted from increased expression of the cell cycle inhibitors in the bulge, since p21^Cip1^, p27^Kip1^, and p57^Kip2^ have been identified as *Hes1* downstream targets in other organs.^32^, ^33^, ^34^ However, our microarray analysis showed that these cell cycle inhibitors are not affected by *Hes1* deletion, but instead Hedgehog signaling is compromised. Notch signaling has been shown to shape the response of neuroepithelial cells to Shh and influences cell fate choice in spinal cord development. Notch activities seem to promote longer primary cilia and ciliary Smo accumulation by an unknown transcriptional mechanism.^35^, ^36^ We found that *Hes1* deletion causes shorter cilia and abolishes further Smo accumulation in the cilia upon Shh treatment, suggesting that *Hes1* does not change the competence but rather the strength of Shh responsiveness during hair growth. Interestingly, Shh emanating from TACs during early anagen has been demonstrated to sustain HF growth and HFSC self‐renewal.^3^ Therefore, our *Hes1* loss‐of‐function studies in HFs suggest that *Hes1* regulates anagen initiation and HF regeneration via modulation of Shh responsiveness. Transcriptome profiling revealed that lipid metabolism is specifically affected in *Hes1*eKO HFSCs. Given that lipid metabolism is closely associated with both Hedgehog signal transduction and Hedgehog ligand modification, the compromised Shh responsiveness caused by *Hes1* deficiency is likely due to altered lipid metabolism that influences ciliogenesis and Smo ciliary accumulation.^37^, ^38^

In telogen HFs, *Gli1* is expressed in two restricted HF epithelial compartments and in the DP. One population of *Gli1*+ cells, localized to the upper margin of the bulge, respond to cutaneous nerve‐releasing Shh, and contribute to wound‐induced epidermal regeneration. Another population of *Gli1*+ cells, localized to the lower portion of bulge/HG, respond to DP/HG‐releasing Shh and contribute to immediate HF growth in anagen.^39^ *Hes1* expression in the lower bulge/HG during anagen initiation suggests a crosstalk between Notch and Hedgehog signaling pathways in this compartment (Figure 7B). Whether Notch signaling promotes or inhibits Hedgehog signaling or vice versa is context‐dependent. Notch receptors and regulated proteolysis enzyme were found to colocalize with cilia. Elimination of primary cilia caused defects in the differentiation of embryonic epidermis, which was attributed to Notch signaling loss.^40^ Normally, Notch receptor is activated by membrane‐bound ligand through cell‐cell interaction but not by soluble forms of ligands, so ciliogenesis is less likely to play a direct role in Notch signaling activation. There are evidences that Shh‐driven stabilization of *Hes1* is independent of canonical Notch signaling and *Hes1* is a Hedgehog‐dependent direct target of *Gli2*.^19^, ^41^, ^42^ In contrast, canonical Notch1/Rbpj axis has been shown to regulate Hedgehog signaling effectors Gli2/Gli3,^43^ as well as *Hes1* is shown to bind the *Gli1* first intron that may inhibit its expression.^44^ Therefore, we think that the crosstalk between Notch and Hedgehog pathways could be different during development, homeostasis, and carcinogenesis.

The two‐step mechanism of SC activation during HF regeneration derives from the observation that HG is in close proximity to the DP and the bulge is separated from the DP by the HG.^45^, ^46^ The DP activates the proliferation of primed SCs in the HG to form the TACs and sustain HF regeneration.^47^ Moreover, the HG is thought to buffer the bulge from the DP to receive excess proliferating signals that will exhaust the conserved SCs. Although the two‐step mode of SC activation seems to prevail as the underlying mechanism of HF regeneration, there are examples that anagen initiation and HF regeneration can occur when Shh signaling is activated in the epithelial part of the HF during telogen,^29^, ^30^ suggesting that ectopic activation of Shh signaling in the bulge can substitute the signal required from the DP to activate the HG. In clinical hair medicine, whether a HF is in refractory or competent telogen^48^ will greatly influence the efficacy of hair growth‐promoting agents. Therefore, perhaps if we can learn more about the alternative modes of HF regeneration then the poorly effective agents can be administered more effectively. Interestingly, Jagged1‐expressing regulatory T cells in the skin are shown to help HFSC activation and anagen induction,^49^ which corroborates our study and suggest that manipulating Notch signaling can be used as a therapeutic strategy to gain control of the telogen stage.

## 5 CONCLUSION

Hedgehog signaling is one of the important pathways that governs epidermal and HF development. A Hedgehog signaling gradient established by the Patched receptors is found along the proximodistal axis of developing HFs,^50^ suggesting that fine‐tuning the intensity of hedgehog signaling is necessary to maintain hair cycle homeostasis. Here, we identified a critical role for *Hes1* in hair cycle homeostasis. By modulating Hedgehog signaling responsiveness, the Notch‐Hes1 axis facilitates signaling activity in the Shh‐receiving HFSCs/HG, which is required for anagen initiation and HFSC maintenance.

## CONFLICT OF INTEREST

The authors indicated no potential conflicts of interest.

## AUTHOR CONTRIBUTIONS

W.‐J.S., S.‐T.L.: collection and/or assembly of data, data analysis and interpretation; L.‐T.Y.: conception and design, data analysis and interpretation, manuscript writing, final approval of manuscript.

## DATA AVAILABILITY STATEMENT

The data that support the findings of this study are available within the article and its supplementary materials.

## Supporting information


**Figure S1 Gross appearance and analysis of hair follicle stem cells and cell death in control and *Hes1*eKO HFs, related to** Figures 1 and 2 **. (A)** Representative pictures of the back skin from control and mutant mice during the postnatal hair cycle. Hair coat of mice was shaved at P20 and growth of the new hair coat was monitored. **(B)** Double immunostaining of Sox9 and CD34 as well as NFATc1 and CD34 in back skin sections at P22. **(C)** Double immunostaining of K15 and CD34 in back skin sections at P22, P24, and P29. **(D)** Quantification of CD34+ and K15+ cells (independent counting) in the bulge at P22, P24 and P29 (mean+/−s.d., > 30 HFs from 2 biological replicates per genotype per stage, n.s.: non‐significant). **(E)** TUNEL staining (arrows) in back skin sections at P19 (catagen) and P22 (telogen). DAPI counterstaining in blue. Bu, bulge; HG, hair germ; DP, derma papillae. Scale bar, 50 μm.

**Figure S2 Analysis of cell death, dermal papilla characteristics, and follicular lineage identity in control and *Hes1*eKO HFs, related to** Figure 2 **. (A)** TUNEL staining in back skin sections at P29 (anagen), P42 (catagen), P56 (telogen). **(B)** Immunostaining of Igfbp3 in back skin sections at P29 and P56. Dotted lines demarcate DP when visible. **(C)** Examination of alkaline phosphatase (AP) activity in the DP of HFs using NBT/BCIP substrate at P22, P24, and P29. **(D)** Immunostaining of Versican in back skin sections at P22, P24, and P29. **(E)** Quantification of Versican+ cells in the DP at P22, P24 and P29 (mean+/−s.d., > 25 HFs from 2 biological replicates per genotype per stage, n.s.: non‐significant). **(F)** Illustration of the hair keratin marker in distinct cell layer of the hair follicle. ORS, outer root sheath; CP, companion layer; *He*, Henle's layer; *Hu*, Huxley's layer; *C*i, cuticle of the *IRS; C*h, cuticle of the hair shaft; Co, cortex of the hair shaft; Me, medulla of the hair shaft. **(G)** K6 immunostaining (arrows) in back skin sections at P29 (anagen). **(H)** Immunostaining analysis of hair keratin markers (AE15, AE13, K82, K73) in back skin sections at P29 and P35. Some sections are double immunostained for Hes1 (arrowheads) to locate *Hes1* expression in the follicular lineages. The arrows mark the positive staining. DAPI counterstaining in blue. Bu, bulge; Bb, hair bulb; DP, derma papillae, Scale bar, 50 μm.

**Figure S3 *Hes1* deficiency causes compromised HF regeneration and HFSC self‐renewal after repetitive depilation, related to** Figure 3 **. (A)** Sequential depilation of control littermate (Ctrl) and *Hes1* conditional knockout (*Hes1*eKO) mice for six rounds with a three‐week interval from the second telogen. Representative pictures of female mice are shown (n = 5). **(B)** Close up of back skin at day 22 post depilation‐induced hair regeneration. **(C)** Back skin sections from repetitive depilation (day 22 post sixth depilation) were double immunostained for CD34 and P‐Cad. **(D)** Quantification of CD34+ bulge and P‐Cad + HG cells in HFs after sequential depilation (mean+/−s.d., n > 50 HFs per genotype from four independent control and mutant pairs, *: *P* < 0.05, ***: *P* < 0.001). **(E)** Close up pictures of the back skin in control and *Hes1*eKO mice at P357.

**Figure S4 Microarray gene expression profiling and bioinformatics analysis on HFSCs from control and *Hes1*eKO mice, related to** Figure 4 **. (A)** Heatmaps and hierarchical clustering, **(B)** Scatter plot, and **(C)** Volcano plot gene expression profile of FACS‐purified HFSCs from two independent control (n = 2) and *Hes1*eKO (n = 2) pairs at P72 (telogen after depilation at P50). Red and green dots delineate upregulated and downregulated genes, respectively. The microarray metadata have been deposited to GEO with the accession number GSE101892 (reviewer access token odcfuqqmjnkhvip). **(D)** Ingenuity pathway analysis result showing the top networks significantly affected by *Hes1* deletion in HFSCs (cut off fold change >1.5 or < −1.5, P < 0.05). Red and green indicate upregulated and downregulated genes in *Hes1*eKO HFSCs, respectively. **(E)** qRT‐PCR analysis of selected genes related to top networks on FACS‐purified HFSCs from control and *Hes1*eKO mice (mean+/−s.d., n = 3 independent control and mutant pairs, *: P < 0.05, **: *P* < 0.01).

**Figure S5 Primary mouse epithelial keratinocyte cultures, related to** Figure 5 **. (A)** Phase contrast photos of primary keratinocyte cultures established from the back skin of newborn control and *Hes1*eKO mice. **(B, C)** Primary keratinocytes were immunostained for K14 and Vimentin to confirm the identity of keratinocytes. Staining only with the secondary antibody served as staining control. **(D)** NIH 3 T3 cells were immunostaining for Vimentin as positive controls for fibroblasts. Scale bar, 100 μm. **(E)** qRT‐PCR analysis of *Hes1* on control and *Hes1*eKO primary keratinocytes. (mean+/−s.d., n = 3 independent experiments, ***: P < 0.001).

**Figure S6 Transient SAG treatment can rescue the HF regeneration in *Hes1*eKO mice after sequential depilation, related to** Figure 6 **. (A)** Representative pictures of the same pair of mice depilated for four rounds from the second telogen with three times of transient application (D1‐D6 post depilation) of vehicle and SAG at the opposite sides of the back skin. **(B)** Bright field images of club hair of four different hair types from control and Hes1eKO mice after SAG recue experiment. Scale bar, 1 mm. **(C)** Quantification of club hair length of each HF type after SAG experiments (mean+/−s.d., n = 20 HFs from each hair types, **: P < 0.01; ***: P < 0.001 determined by ANOVA).

**Appendix S1**: Supplementary Materials and Methods.
