# Supplementary figures and images for: Hes1 regulates anagen initiation and hair follicle regeneration through modulation of hedgehog signaling
Source: Stem Cells. 2019 Nov 26;38(2):301–14. doi: 10.1002/stem.3117 (PMC7027765; doi:10.1002/stem.3117)

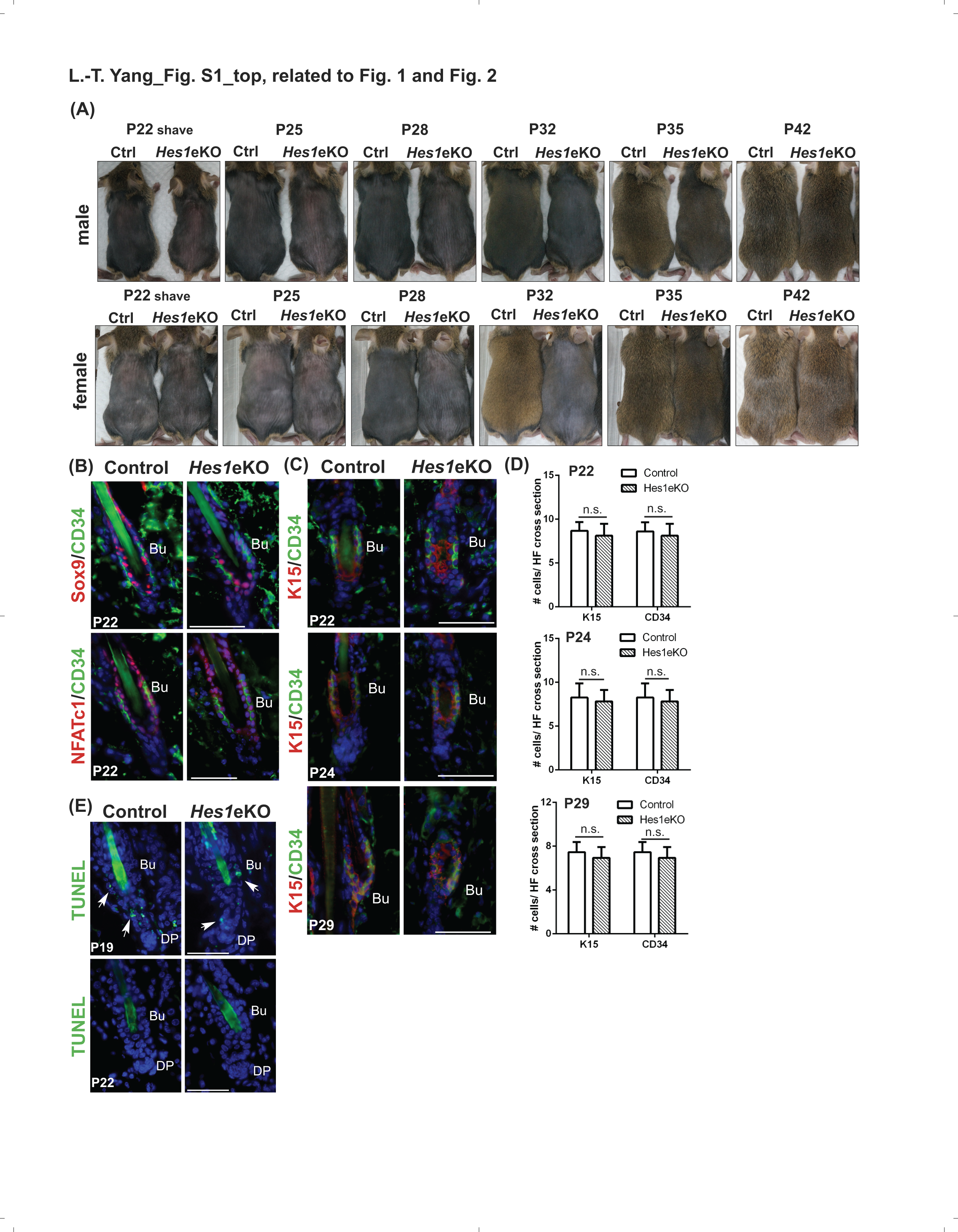

Supplement: Supplementary file 1 — Figure S1 Gross appearance and analysis of hair follicle stem cells and cell death in control and Hes1eKO HFs, related to Figures 1 and 2 . (A) Representative pictures of the back skin from control and mutant mice during the postnatal hair cycle. Hair coat of mice was shaved at P20 and growth of the new hair coat was monitored. (B) Double immunostaining of Sox9 and CD34 as well as NFATc1 and CD34 in back skin sections at P22. (C) Double immunostaining of K15 and CD34 in back skin sections at P22, P24, and P29. (D) Quantification of CD34+ and K15+ cells (independent counting) in the bulge at P22, P24 and P29 (mean+/−s.d., > 30 HFs from 2 biological replicates per genotype per stage, n.s.: non‐significant). (E) TUNEL staining (arrows) in back skin sections at P19 (catagen) and P22 (telogen). DAPI counterstaining in blue. Bu, bulge; HG, hair germ; DP, derma papillae. Scale bar, 50 μm. [file STEM-38-301-s001.tif]

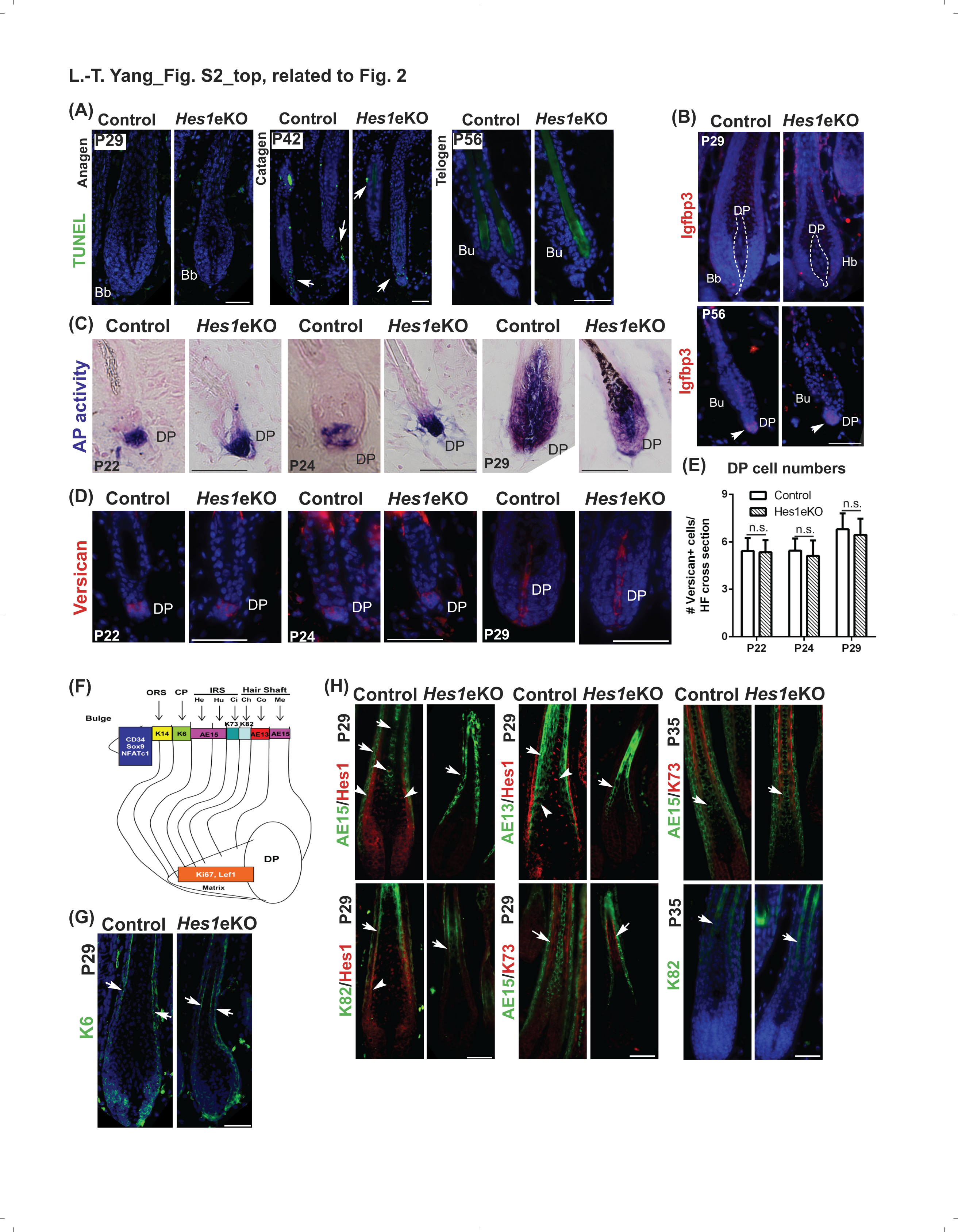

Supplement: Supplementary file 2 — Figure S2 Analysis of cell death, dermal papilla characteristics, and follicular lineage identity in control and Hes1eKO HFs, related to Figure 2 . (A) TUNEL staining in back skin sections at P29 (anagen), P42 (catagen), P56 (telogen). (B) Immunostaining of Igfbp3 in back skin sections at P29 and P56. Dotted lines demarcate DP when visible. (C) Examination of alkaline phosphatase (AP) activity in the DP of HFs using NBT/BCIP substrate at P22, P24, and P29. (D) Immunostaining of Versican in back skin sections at P22, P24, and P29. (E) Quantification of Versican+ cells in the DP at P22, P24 and P29 (mean+/−s.d., > 25 HFs from 2 biological replicates per genotype per stage, n.s.: non‐significant). (F) Illustration of the hair keratin marker in distinct cell layer of the hair follicle. ORS, outer root sheath; CP, companion layer; He, Henle's layer; Hu, Huxley's layer; Ci, cuticle of the IRS; Ch, cuticle of the hair shaft; Co, cortex of the hair shaft; Me, medulla of the hair shaft. (G) K6 immunostaining (arrows) in back skin sections at P29 (anagen). (H) Immunostaining analysis of hair keratin markers (AE15, AE13, K82, K73) in back skin sections at P29 and P35. Some sections are double immunostained for Hes1 (arrowheads) to locate Hes1 expression in the follicular lineages. The arrows mark the positive staining. DAPI counterstaining in blue. Bu, bulge; Bb, hair bulb; DP, derma papillae, Scale bar, 50 μm. [file STEM-38-301-s002.tif]

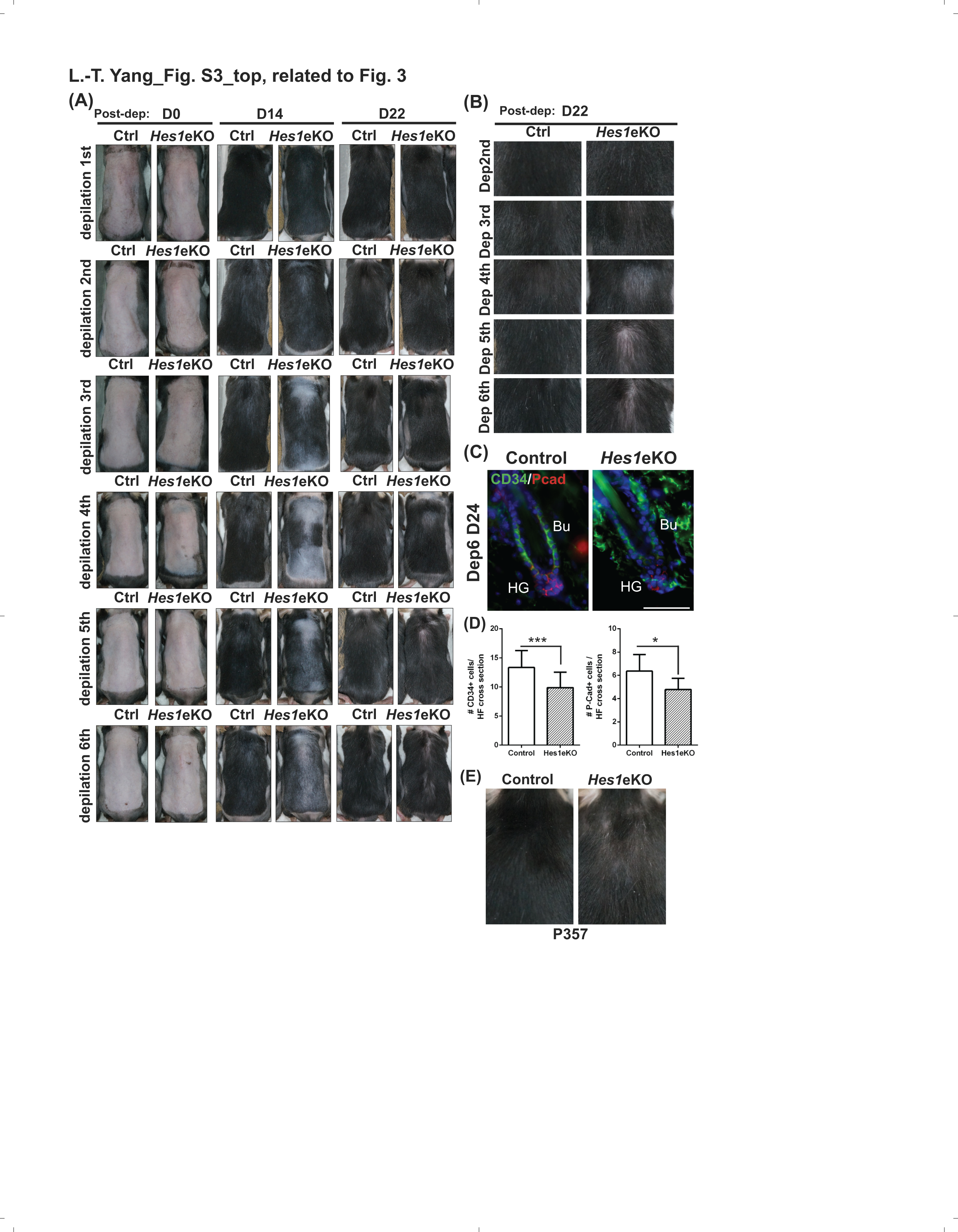

Supplement: Supplementary file 3 — Figure S3 Hes1 deficiency causes compromised HF regeneration and HFSC self‐renewal after repetitive depilation, related to Figure 3 . (A) Sequential depilation of control littermate (Ctrl) and Hes1 conditional knockout (Hes1eKO) mice for six rounds with a three‐week interval from the second telogen. Representative pictures of female mice are shown (n = 5). (B) Close up of back skin at day 22 post depilation‐induced hair regeneration. (C) Back skin sections from repetitive depilation (day 22 post sixth depilation) were double immunostained for CD34 and P‐Cad. (D) Quantification of CD34+ bulge and P‐Cad + HG cells in HFs after sequential depilation (mean+/−s.d., n > 50 HFs per genotype from four independent control and mutant pairs, *: P < 0.05, ***: P < 0.001). (E) Close up pictures of the back skin in control and Hes1eKO mice at P357. [file STEM-38-301-s003.tif]

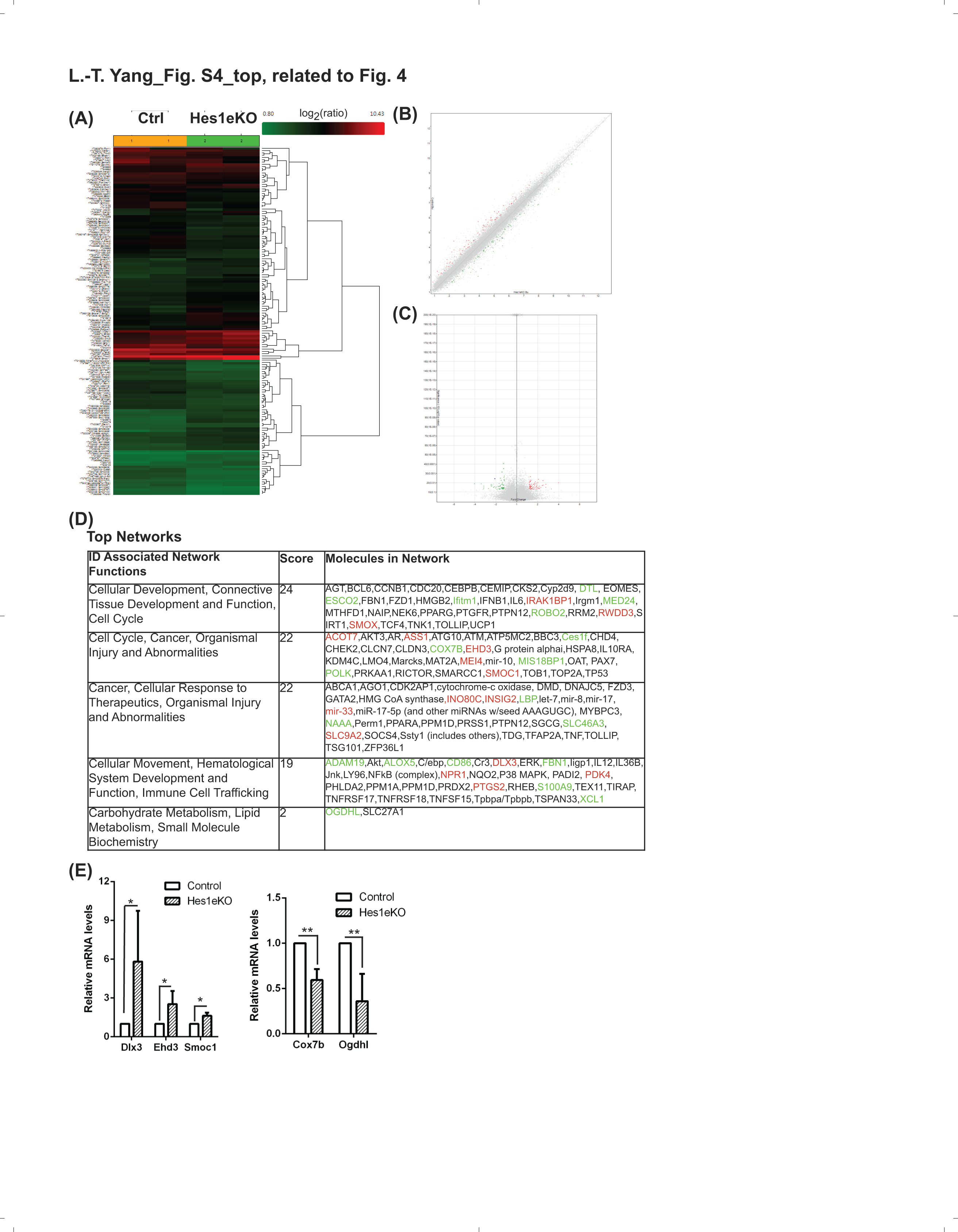

Supplement: Supplementary file 4 — Figure S4 Microarray gene expression profiling and bioinformatics analysis on HFSCs from control and Hes1eKO mice, related to Figure 4 . (A) Heatmaps and hierarchical clustering, (B) Scatter plot, and (C) Volcano plot gene expression profile of FACS‐purified HFSCs from two independent control (n = 2) and Hes1eKO (n = 2) pairs at P72 (telogen after depilation at P50). Red and green dots delineate upregulated and downregulated genes, respectively. The microarray metadata have been deposited to GEO with the accession number GSE101892 (reviewer access token odcfuqqmjnkhvip). (D) Ingenuity pathway analysis result showing the top networks significantly affected by Hes1 deletion in HFSCs (cut off fold change >1.5 or < −1.5, P < 0.05). Red and green indicate upregulated and downregulated genes in Hes1eKO HFSCs, respectively. (E) qRT‐PCR analysis of selected genes related to top networks on FACS‐purified HFSCs from control and Hes1eKO mice (mean+/−s.d., n = 3 independent control and mutant pairs, *: P < 0.05, **: P < 0.01). [file STEM-38-301-s004.tif]

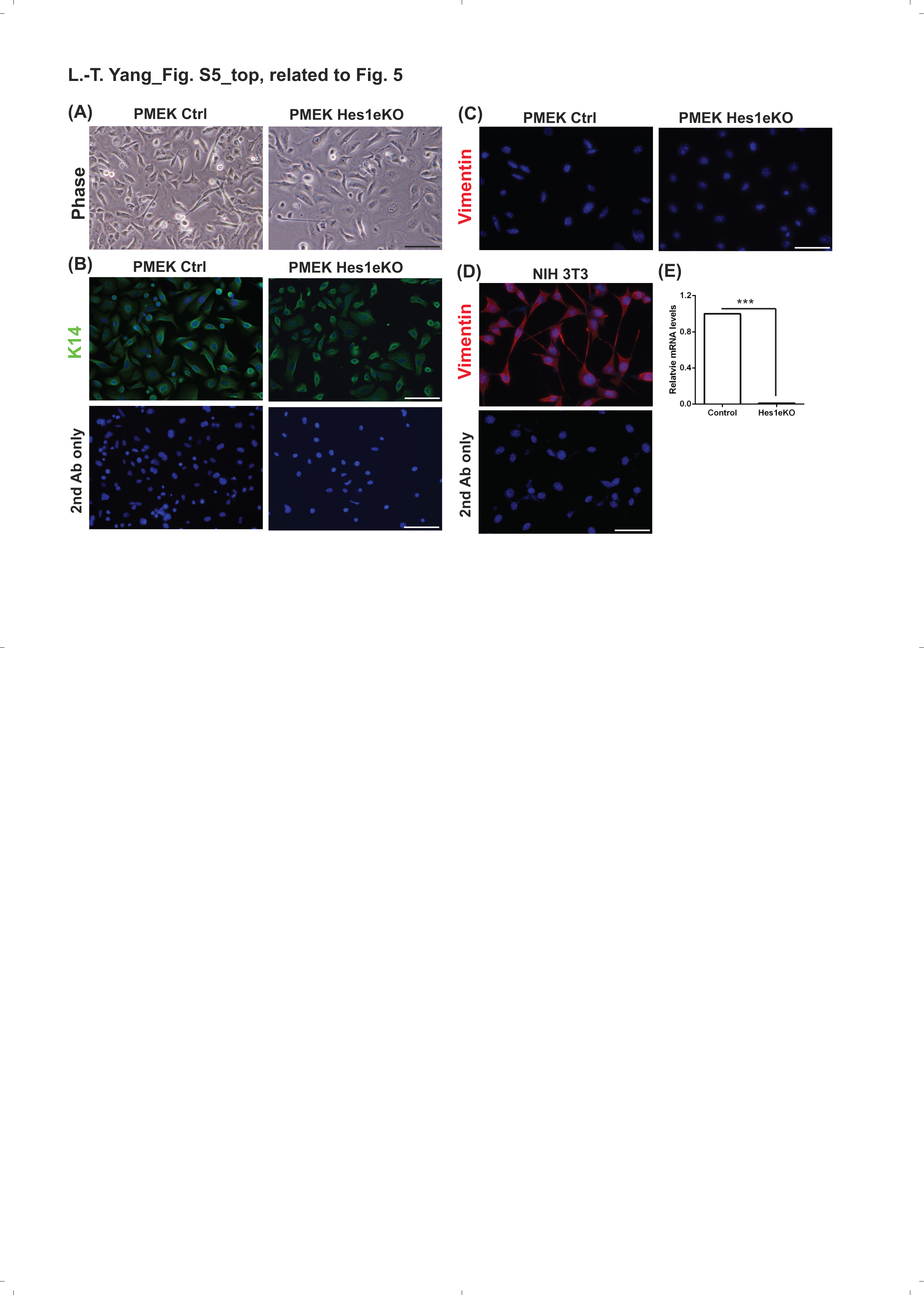

Supplement: Supplementary file 5 — Figure S5 Primary mouse epithelial keratinocyte cultures, related to Figure 5 . (A) Phase contrast photos of primary keratinocyte cultures established from the back skin of newborn control and Hes1eKO mice. (B, C) Primary keratinocytes were immunostained for K14 and Vimentin to confirm the identity of keratinocytes. Staining only with the secondary antibody served as staining control. (D) NIH 3 T3 cells were immunostaining for Vimentin as positive controls for fibroblasts. Scale bar, 100 μm. (E) qRT‐PCR analysis of Hes1 on control and Hes1eKO primary keratinocytes. (mean+/−s.d., n = 3 independent experiments, ***: P < 0.001). [file STEM-38-301-s005.tif]

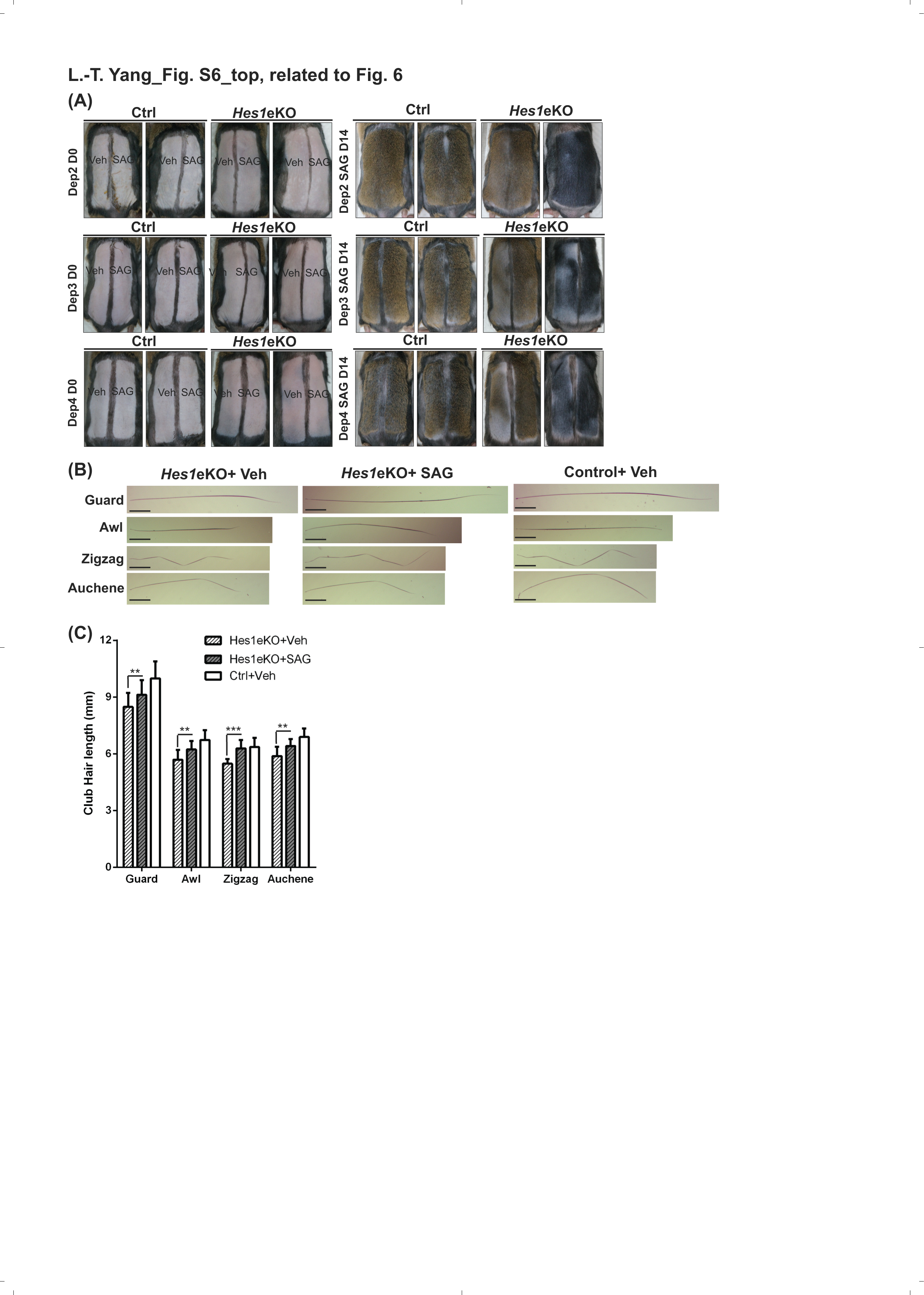

Supplement: Supplementary file 6 — Figure S6 Transient SAG treatment can rescue the HF regeneration in Hes1eKO mice after sequential depilation, related to Figure 6 . (A) Representative pictures of the same pair of mice depilated for four rounds from the second telogen with three times of transient application (D1‐D6 post depilation) of vehicle and SAG at the opposite sides of the back skin. (B) Bright field images of club hair of four different hair types from control and Hes1eKO mice after SAG recue experiment. Scale bar, 1 mm. (C) Quantification of club hair length of each HF type after SAG experiments (mean+/−s.d., n = 20 HFs from each hair types, **: P < 0.01; ***: P < 0.001 determined by ANOVA). [file STEM-38-301-s006.tif]
