# Supplementary material for: Hes1 regulates anagen initiation and hair follicle regeneration through modulation of hedgehog signaling
Source: Stem Cells. 2019 Nov 26;38(2):301–14. doi: 10.1002/stem.3117 (PMC7027765; doi:10.1002/stem.3117)
Supplement: Supplementary file 7 — Appendix S1: Supplementary Materials and Methods. [file STEM-38-301-s007.docx]

**Supplementary materialS**

**Supplementary Figure Legends**

**Figure S1. Gross appearance and analysis of hair follicle stem cells and cell death in control and *Hes1*eKO HFs, related to figure 1 and 2. (A)** Representative pictures of the back skin from control and mutant mice during the postnatal hair cycle. Hair coat of mice was shaved at P20 and growth of the new hair coat was monitored. **(B)** Double immunostaining of Sox9 and CD34 as well as NFATc1 and CD34 in back skin sections at P22. **(C)** Double immunostaining of K15 and CD34 in back skin sections at P22, P24, and P29. **(D)** Quantification of CD34+ and K15+ cells (independent counting) in the bulge at P22, P24 and P29 (mean+/-s.d., > 30 HFs from 2 biological replicates per genotype per stage, n.s.: non-significant). **(E)** TUNEL staining (arrows) in back skin sections at P19 (catagen) and P22 (telogen). DAPI counterstaining in blue. Bu, bulge; HG, hair germ; DP, derma papillae. Scale bar, 50 μm.

**Figure S2.** **Analysis of cell death, dermal papilla characteristics, and follicular lineage identity in control and *Hes1*eKO HFs, related to figure 2. (A)** TUNEL staining in back skin sections at P29 (anagen), P42 (catagen), P56 (telogen). **(B)** Immunostaining of Igfbp3 in back skin sections at P29 and P56. Dotted lines demarcate DP when visible. **(C)** Examination of alkaline phosphatase (AP) activity in the DP of HFs using NBT/BCIP substrate at P22, P24, and P29. **(D)** Immunostaining of Versican in back skin sections at P22, P24, and P29. **(E)** Quantification of Versican+ cells in the DP at P22, P24 and P29 (mean+/-s.d., > 25 HFs from 2 biological replicates per genotype per stage, n.s.: non-significant). **(F)** Illustration of the hair keratin marker in distinct cell layer of the hair follicle. ORS, outer root sheath; CP, companion layer; He, Henle’s layer; Hu, Huxley’s layer; Ci, cuticle of the IRS; Ch, cuticle of the hair shaft; Co, cortex of the hair shaft; Me, medulla of the hair shaft. **(G)** K6 immunostaining (arrows) in back skin sections at P29 (anagen). **(H)** Immunostaining analysis of hair keratin markers (AE15, AE13, K82, K73) in back skin sections at P29 and P35. Some sections are double immunostained for Hes1 (arrowheads) to locate *Hes1* expression in the follicular lineages. The arrows mark the positive staining. DAPI counterstaining in blue. Bu, bulge; Bb, hair bulb; DP, derma papillae, Scale bar, 50 μm.

**Figure S3.** ***Hes1* deficiency causes compromised HF regeneration and HFSC self-renewal after repetitive depilation, related to figure 3. (A)** Sequential depilation of control littermate (Ctrl) and *Hes1* conditional knockout (*Hes1*eKO) mice for six rounds with a three-week interval from the second telogen. Representative pictures of female mice are shown (n = 5). **(B)** Close up of back skin at day 22 post depilation-induced hair regeneration. **(C)** Back skin sections from repetitive depilation (day 22 post 6^th^ depilation) were double immunostained for CD34 and P-Cad. **(D)** Quantification of CD34+ bulge and P-Cad+ HG cells in HFs after sequential depilation (mean+/-s.d., n > 50 HFs per genotype from four independent control and mutant pairs, *: P<0.05, ***: P<0.001). **(E)** Close up pictures of the back skin in control and *Hes1*eKO mice at P357.

**Figure S4.** **Microarray gene expression profiling and bioinformatics analysis on HFSCs from control and *Hes1*eKO mice, related to figure 4. (A)** Heatmaps and hierarchical clustering, **(B)** Scatter plot, and **(C)** Volcano plot gene expression profile of FACS-purified HFSCs from two independent control (n = 2) and *Hes1*eKO (n = 2) pairs at P72 (telogen after depilation at P50). Red and green dots delineate upregulated and downregulated genes, respectively. The microarray metadata have been deposited to GEO with the accession number GSE101892 (reviewer access token odcfuqqmjnkhvip). **(D)** Ingenuity pathway analysis result showing the top networks significantly affected by *Hes1* deletion in HFSCs (cut off fold change >1.5 or <-1.5, P<0.05). Red and green indicate upregulated and downregulated genes in *Hes1*eKO HFSCs, respectively. **(E)** qRT-PCR analysis of selected genes related to top networks on FACS-purified HFSCs from control and *Hes1*eKO mice (mean+/-s.d., n = 3 independent control and mutant pairs, *: P<0.05, **: P<0.01).

**Figure S5. Primary mouse epithelial keratinocyte cultures, related to figure 5. (A)** Phase contrast photos of primary keratinocyte cultures established from the back skin of newborn control and *Hes1*eKO mice. **(B, C)** Primary keratinocytes were immunostained for K14 and Vimentin to confirm the identity of keratinocytes. Staining only with the secondary antibody served as staining control. **(D)** NIH 3T3 cells were immunostaining for Vimentin as positive controls for fibroblasts. Scale bar, 100 μm. **(E)** qRT-PCR analysis of *Hes1* on control and *Hes1*eKO primary keratinocytes. (mean+/-s.d., n = 3 independent experiments, ***: P<0.001).

**Figure S6.** **Transient SAG treatment can rescue the HF regeneration in *Hes1*eKO mice after sequential depilation, related to figure 6. (A)** Representative pictures of the same pair of mice depilated for four rounds from the second telogen with three times of transient application (D1-D6 post depilation) of vehicle and SAG at the opposite sides of the back skin. **(B)** Bright field images of club hair of four different hair types from control and Hes1eKO mice after SAG recue experiment. Scale bar, 1 mm. **(C)** Quantification of club hair length of each HF type after SAG experiments (mean+/-s.d., n = 20 HFs from each hair types, **: P<0.01; ***: P<0.001 determined by ANOVA).

**Supplementary Materials and Methods**

**EdU Labeling, Cell Proliferation, Cell Death, and Biochemical Analyses**

For EdU labeling, mice were injected intraperitoneally with EdU (Jena Bioscience GmbH) at 50 μg/g body weight and sacrificed 3 hours later. Cell proliferation analysis was done by Click-iT EdU Alex Fluor 594 Imaging kit (Thermo Fisher Scientific) following the manufacturer’s protocol. TUNEL assays were conducted using the DeadEnd Fluorometric TUNEL system (Promega) following the manufacturer’s instruction. The NAD^+^/NADH ratio is measured using the NAD/NADH Quantitation Colorimetric Kit (Biovision) following the manufacture’s protocol.

**RNA Isolation and Quantitative Real-Time PCR (qRT-PCR)**

Total RNA was isolated using TRIzol (Invitrogen), followed by purification using RNeasy Mini Kit (Qiagen) according to the manufacturer’s protocol. Equal amounts (~ 2 μg) of RNA were reverse-transcribed using Transcriptor Reverse Transcriptase (Roche) and oligo-dT primers following the manufacturer’s instruction. Real-time PCR was conducted using an ABI 7500 Real-Time PCR system (Applied Biosystems, Foster City) with FastStart Universal Probe Master (Rox, Roche) and probe/primer sets (Roche Universal Probe). Samples were analyzed by SDS Software v1.4, calculated based on the formula 2^–ΔΔCP^, and normalized to the housekeeping genes Rpl7. Gene-specific universal probe numbers and primer sequences are available upon request. Total RNA isolated from bulge keratinocytes was amplified by the REPLI-g WTA single Cell Kit (Qiagen) to obtain sufficient amount for qRT-PCR analysis.

**Microarray and Bioinformatics Analysis**

HFSC RNAs from FACS-purified control and *Hes1*eKO mice were sent to NHRI Microarray Core Facility for determination of RNA quality and concentrations using RNA 6000 Pico Assay on Agilent 2100 Bioanalyzer (Agilent Technologies). High quality RNA (RIN > 8) was amplified, hybridized with GeneChip^TM^ Mouse gene 2.0 ST Arrays (Affymetrix), and scanned using a GeneChip®Scanner 3000. Two independent control (n=2) and mutant (n=2) sample pairs were used for statistical analysis by Partek Genomics Suite software (PGS, v6.6, Partek, Inc.). Probes sets were identified as differentially expressed by setting the cutoff fold change >1.5 or <-1.5 (P <0.05).

Heatmaps, scatter plot, and hierarchical clustering of microarray metadata (GEO# GSE101892) were generated using the Transcriptome Analysis Console 3.0 software (Affymetrix). The ingenuity pathway analysis (Qiagen Bioinformatics) was used to analyze the genes significantly affected by *Hes1* deletion (cut off fold change >1.5 or <-1.5, P <0.05). GSEA analysis was performed using the javaGSEA Desktop Application [1] with 1,000 times of permutation in gene sets. For a gene with multiple probes, the probe with a fold change of most extreme value was selected as the input. The log2R was used as input metrics for the enrichment analyses, where R is the ratio of gene expression level in control to that in knockout samples. Several gene set collections in MSigDB, including canonical pathways (C2-CP), biological process gene sets (C5-BP), and hallmark gene sets v5.0 were separately used as target gene sets. Gene set with a false discovery rate (FDR) ≤ 0.25 or nominal P value <0.05 is considered to be enriched significantly.

**Reporter Assay**

To measure Hedgehog signaling activation, mouse primary keratinocytes were cotransfected with 200 ng of either 8x3’Gli-BS or 8xm3’Gli-BS firefly luciferase reporter plasmid [2] along with 5 ng of pRLTK Renilla luciferase reporter plasmid (Promega) in a 12-well plate using TransIT®-2020 Transfection Reagent (Mirus Bio LLC). After 16 hours, cells were serum starved (0.1% chelexed-FBS) for ciliated cell enrichment and treated with either vehicle, 20 nM SAG, or 20 nM Shh-N (R&D) for an additional 20 hours. Cells were harvested in passive lysis buffer and assayed using dual-luciferase reporter assay system (Promega) following the manufacture’s protocol. The normalized luciferase activity represents the ratio of 8x3’Gli-BS firefly/pRLTK Renilla activity to 8xm3’Gli-BS firefly/pRLTK Renilla activity.

**Supplementary References**

1 Subramanian A, Tamayo P, Mootha VK, et al. Gene set enrichment analysis: a knowledge-based approach for interpreting genome-wide expression profiles. Proc Natl Acad Sci U S A*.* 2005;102:15545-15550.

2 Sasaki H, Hui C, Nakafuku M, et al. A binding site for Gli proteins is essential for HNF-3beta floor plate enhancer activity in transgenics and can respond to Shh in vitro. Development*.* 1997;124:1313-1322.
